# Obesity measures, metabolic health and their association with 15-year all-cause and cardiovascular mortality in the SAMINOR 1 Survey: a population-based cohort study

**DOI:** 10.1186/s12872-021-02288-9

**Published:** 2021-10-21

**Authors:** Vilde Lehne Michalsen, Sarah H. Wild, Kirsti Kvaløy, Johan Svartberg, Marita Melhus, Ann Ragnhild Broderstad

**Affiliations:** 1 Quality and Research Department, University Hospital of North Norway, Tromsø, Norway; 2 Centre for Sami Health Research, Department of Community Medicine, Faculty of Health Sciences, UiT The Arctic University of Norway, 9037 Tromsø, Norway; 3 Usher Institute, University of Edinburgh, Edinburgh, Scotland, UK; 4 Department of Public Health and Nursing, Norwegian University of Science and Technology (NTNU), Trondheim, Norway; 5 Division of Internal Medicine, University Hospital of North Norway, Tromsø, Norway; 6 Tromsø Endocrine Research Group, Department of Clinical Medicine, UiT The Arctic University of Norway, Tromsø, Norway; 7 Division of Internal Medicine, Department of Medicine, University Hospital of North Norway, Harstad, Norway

**Keywords:** Abdominal obesity, A body shape index, All-cause mortality, Body mass index, Cardiovascular mortality, Metabolically healthy obesity, Metabolic syndrome, Obesity, Waist circumference

## Abstract

**Background:**

The mortality of metabolic-obesity phenotypes has been thoroughly studied, but it is not known if or how the association between mortality and body mass index (BMI), waist circumference or a body shape index (ABSI) differ in strata of cardiometabolic health status.

**Methods:**

We linked data on 12,815 men and women aged 36–79 years from the SAMINOR 1 Survey with mortality data from the Norwegian Cause of Death Registry. We defined metabolically healthy and unhealthy as having zero and ≥ 1, respectively, of the following: MetS, pre-existing diabetes or cardiovascular disease (CVD), or prescribed drugs for high blood pressure, hyperglycaemia or dyslipidaemia. We defined general and abdominal obesity as BMI ≥ 30 kg/m^2^ and waist circumference ≥ 88 cm (women) or 102 cm (men), respectively, and cross-classified these categories with metabolic status to create metabolically healthy non-obese and obese (MHNO and MHO) and metabolically unhealthy non-obese and obese (MUNO and MUO) phenotypes. We used Cox regression to estimate the hazard ratio (HR) for all-cause and CVD mortality for 1) the four phenotypes and 2) BMI, waist circumference and ABSI fitted with restricted cubic splines. We adjusted for age and lifestyle, and tested for interactions with sex and metabolic status (only continuous measures).

**Results:**

The MHO phenotype was present in 7.8% of women and 5.8% of men. During a median follow-up of 15.3/15.2 years, 596/938 women/men had died, respectively. The MUNO and MUO groups had higher mortality than the MHNO group. Sex and phenotypes interacted with respect to CVD mortality: relative to the MHNO group, the MHO group had an adjusted HR (95% confidence interval) for CVD mortality of 1.05 (0.38–2.88) in women and 2.92 (1.71–5.01) in men. We found curvilinear associations between BMI/waist circumference and all-cause mortality irrespective of metabolic status. Corresponding relationships with CVD mortality were linear and the slope differed by sex and metabolic status. ABSI was linearly and positively associated with all-cause and CVD mortality in men.

**Conclusion:**

The relationships between BMI, waist circumference or ABSI and mortality differed by sex, metabolic status and cause of death. Poor metabolic health substantially increases mortality regardless of obesity status.

**Supplementary Information:**

The online version contains supplementary material available at 10.1186/s12872-021-02288-9.

## Background

The prevalence of obesity doubled between 1980 and 2015 in more than 70 countries [1]. Obesity is a strong driver of a cluster of risk factors known as metabolic syndrome (MetS). MetS is etiologically linked to insulin resistance and visceral adipose tissue that promotes a proinflammatory and prothrombotic state, making it an antecedent of both cardiovascular disease (CVD) and type 2 diabetes mellitus [2]. At least half of the cardiovascular risk linked to obesity is mediated through metabolic risk factors [3, 4]. In Europe, approximately 7–19% of people with obesity do not have MetS, so-called metabolically healthy obesity (MHO) [5]. Accumulating evidence strongly suggests that, compared to the metabolically healthy normal-weight group, people with MHO are at increased risk of CVD [6–8], type 2 diabetes mellitus [9, 10], and mortality [11, 12].

A body mass index (BMI) ≥ 30 kg/m^2^ is commonly used to define obesity in populations of European ancestry, but BMI is a crude marker of body fat distribution. Waist circumference is a better measure of the visceral adipose tissue that is particularly strongly associated with cardiometabolic disease [13]. BMI and waist circumference usually show J- or U-shaped associations with mortality [14, 15]. This may indicate a functional relationship not reflected well by crude dichotomies, as dichotomisation of continuous predictors cause loss of information and statistical power to demonstrate associations [16]. However, BMI and waist circumference are usually highly correlated. Krakauer et al. developed a body shape index (ABSI), which is a measure of central obesity that has a low correlation with BMI [17].

To the best of our knowledge, no studies have examined the relationships between continuous measures of BMI, waist circumference or ABSI and mortality by metabolic health status. We aimed to examine these relationships using a population-based multi-ethnic sample of adult women and men from rural Northern Norway, which has high prevalence of both general and abdominal obesity and MetS [18, 19].

## Methods

### Data

We used the national 11-digit personal identity number linking individual data from the three following sources: baseline information on participants in the SAMINOR 1 Survey (the first survey of the Population-based Study on Health and Living Conditions in Regions with Sami and Norwegian Populations—the SAMINOR Study), mortality data from the Norwegian Cause of Death Registry, and information on emigration from Statistics Norway.

The population of Northern Norway includes people of Norwegian, Sami and Kven (descendants of Finnish immigrants in the 18th and 19th Century) ethnicity. The Sami is an ethnic minority and acknowledged as an indigenous people. Traditionally, the Sami inhabited Northern parts of Norway, Sweden, Finland and the Kola Peninsula in the Russian Federation.

The SAMINOR Study is a population-based study designed to investigate the health and living conditions in regions of Norway with an assumed proportion of at least 5–10% Sami inhabitants. The Centre for Sami Health Research at UiT The Arctic University of Norway and the Norwegian Institute of Public Health conducted the SAMINOR 1 Survey in 2003–2004 in 24 rural municipalities mainly in northern parts of Norway. Clinical measurements, blood samples and self-administered questionnaire data were collected on men and women aged 36–79 years. Of 27,151 invited individuals, 16,455 (60.6%) participated and consented to have their data linked to medical and national registries. Survey details have been reported previously [20].

### Clinical measurements

The following measurements of each participant were made by trained personnel: waist circumference, recorded to the nearest centimetre at the umbilicus, the participant standing and breathing normally; height and weight, measured to the nearest 0.1 cm and 100 g, respectively, using an electronic scale with participants wearing light clothing and no shoes; and blood pressure, measured with a Dinamap‐R automatic device (Critikon, Tampa, Florida, USA). Blood pressure was measured after a 2‐minute seated rest, and three measurements with 1‐minute intervals were recorded. The first measurement was discarded and the average of the second and third was used. Trained personnel performed venepuncture with the participant in a seated position and non-fasting blood samples were centrifuged within 1.5 h. Serum was sent by overnight post to the laboratory at Ullevål University Hospital, Oslo. Lipids and glucose were measured by an enzymatic method (Hitachi 917 autoanalyzer, Roche Diagnostic, Switzerland).

### Lifestyle and disease variables

Participants were asked to fill in a questionnaire from which we obtained the following information (answer options in parenthesis): education (total number of school years); diabetes (yes/no); angina pectoris (yes/no); previous stroke (yes/no); previous heart attack (yes/no); use of blood pressure-lowering drug (currently/previously, but not now/never); use of cholesterol‐lowering drug (currently/previously, but not now/never); use of insulin (currently/previously, but not now/never); use of glucose‐lowering drug in tablet format (currently/previously, but not now/never); smoking (currently/previously/never); alcohol consumption (never/not this year/a few times during this year/1 time per month/2–3 times per month/1 time per week/2–3 times per week/4‐7 times per week). Alcohol consumption was categorised into “weekly alcohol consumption”, “less than weekly alcohol consumption” and “never/not last year”. Leisure‐time physical activity was measured by a self-reported modified Saltin-Grimby Physical Activity Level scale (reading, watching television, or engaging in sedentary activities/at least 4 h a week of walking, bicycling, or other types of physical activity/at least 4 h a week of participating in recreational athletics or heavy gardening/regular, vigorous training or participating in competitive sports several times a week) [21]. The Saltin-Grimby Physical Activity Level scale has been used in many Nordic populations and has shown acceptable validity regarding objectively measured physical activity [21]. Leisure-time physical activity was categorised into “sedentary” (the first option), “light” (the second option) and “moderate-hard” (the last two options merged). Participants were also asked to list any medication they had used within the last four weeks and the information was combined with information from drug-specific questions, details are found elsewhere [22].

The questionnaire also included questions (11 in total) on use of language at home by grandparents, parents and participants, ethnic background for parents and participants, and the participants’ self‐perceived ethnicity (one or more of these alternatives were allowed: Norwegian, Sami, Kven, and other). Participants were categorised as Sami if they answered Sami as (1) their self-perceived ethnicity or (2) their own ethnic background. All others were categorised as non-Sami.

### Independent variables

We defined MetS according to the ‘harmonised’ Adult Treatment Panel-III definition, with some adaptations [23]. At least three of the following five components had to be present:

hypertension, defined as systolic blood pressure ≥ 130 mmHg or diastolic blood pressure ≥ 85 mmHg or current use of antihypertensive drug;

elevated random glucose, defined as random serum glucose ≥ 7.8 mmol/L or self-reported diabetes;

increased waist circumference, defined as waist circumference ≥ 80 cm in women and ≥ 94 cm in men;

hypertriglyceridemia, defined as random serum triglycerides ≥ 1.7 mmol/L; and

lowered HDL cholesterol, defined as random serum HDL cholesterol < 1.3 mmol/L in women and < 1.0 mmol/L in men.

Participants were categorised as metabolically unhealthy if they had any of the following, as recommended by Smith et al. [24]:

MetS (for abdominal obesity phenotypes, the MetS definition was modified to the presence of any given two or more components excluding increased waist circumference);

self-reported diabetes, stroke, angina pectoris, or myocardial infarction;

self-reported current treatment for high blood pressure, hyperglycaemia or dyslipidaemia.

General and abdominal obesity were defined as BMI ≥ 30 kg/m^2^ and waist circumference ≥ 88 cm in women and ≥ 102 cm in men, respectively. The following general obesity phenotypes were created: metabolically healthy non-obesity (MHNO); metabolically unhealthy non-obesity (MUNO); metabolically healthy obesity (MHO); and metabolically unhealthy obesity (MUO). The following abdominal obesity phenotypes were created: metabolically healthy non-abdominal-obesity (MHNAO); metabolically unhealthy non-abdominal-obesity (MUNAO); metabolically healthy abdominal obesity (MHAO); and metabolically unhealthy abdominal obesity (MUAO).

In addition to using BMI and waist circumference to define general and abdominal obesity, respectively, we also used them as continuous variables (BMI in kg/m^2^ and waist circumference in cm). Due to the high correlation between BMI and waist circumference (0.88 in women and 0.86 in men in this cohort), we also applied ABSI as developed by Krakauer et al. based on a U.S. population-based cohort (NHANES) [17]:

$$ABSI= \frac{waist circumference}{{{BMI}^{2/3} height}^{1/2}}$$

The ABSI was transformed to a Z-score for interpretability by subtracting the sex-specific mean and dividing by the sex-specific standard deviation. ABSI was not used as a determinant of categorical obesity because of the lack of validated cut-offs.

### Outcome variables

Mortality data comprised date of death and underlying cause of death, coded using the International Statistical Classification of Diseases and Related Health Problems, 10^th^ revision. The study period started at the date of study entry (between 14th January 2003 and 5th March 2004) and ended at date of death (the event), date of emigration (censored) or the end of follow-up 31st December 2018 (censored), whichever occurred first. The outcome variables of interest were all-cause mortality and CVD mortality (death from causes I00-I99).

### Missing data and exclusions

**Fig. 1:**
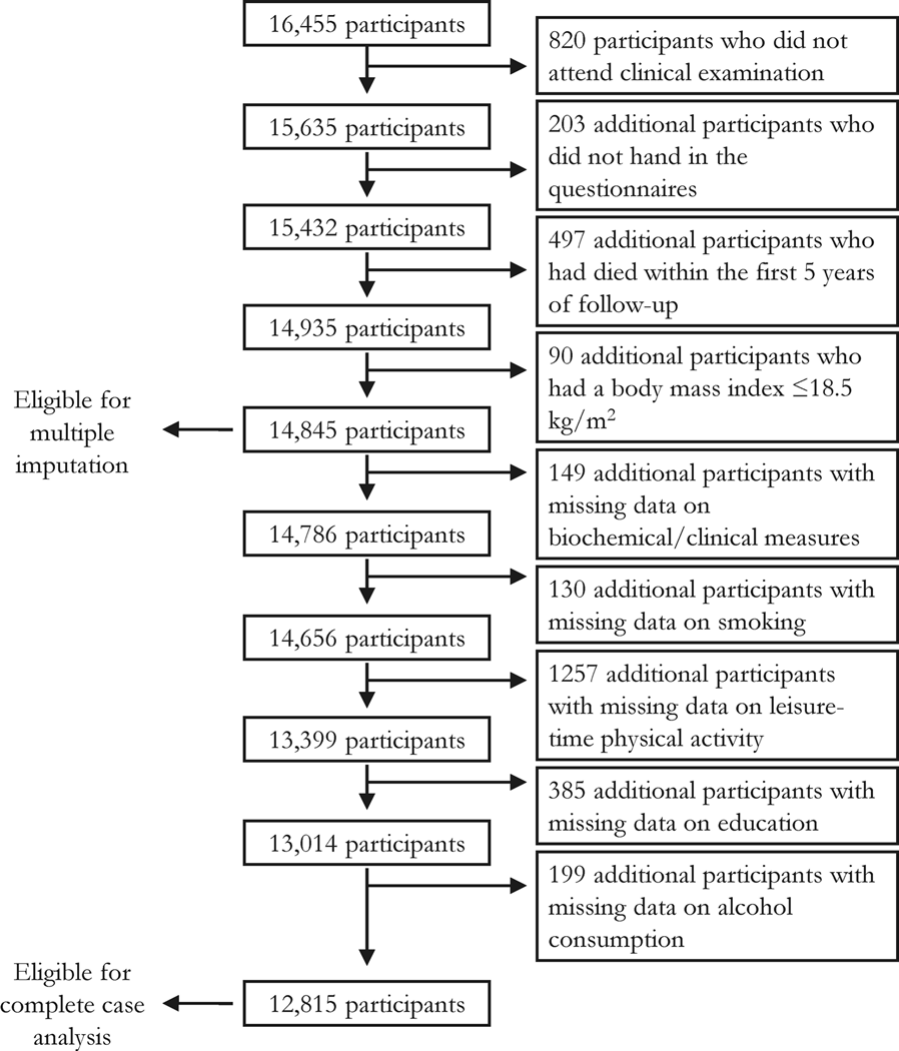
Flow-chart describing cohort selection from SAMINOR 1 participants and patterns of missing data

Figure 1 shows a flow chart describing the cohort selection. We excluded 497 participants who died within the first 5 years of follow-up and 90 participants with a BMI ≤ 18.5 kg/m^2^ to avoid the potential for reverse causality [14]. Because information on pre-existing disease or prescribed drugs was not necessary for the categorisation, we did not exclude participants with missing data for these variables. However, most participants with missing data for these variables were categorised into a metabolically unhealthy group by other determinants (Table 1). After exclusions, the complete case analytical sample comprised 12,815 participants, 47.2% of the invited sample.

**Table.1:** Sample characteristics in mean (standard deviation) or frequency (percent) according to general obesity phenotypes in 6517 women in the SAMINOR 1 Survey (2003–2004)

	Metabolically healthy non-obesity (N = 3095, 47.5%)	Metabolically unhealthy non-obesity (N = 1662, 25.5%)	Metabolically healthy obesity (N = 510, 7.8%)	Metabolically unhealthy obesity (N = 1250, 19.2%)	Total (N = 6517)	*p* value
**Age (years)**	49.4 (9.4)	57.4 (10.7)	52.1 (10.2)	57.4 (11.0)	53.2 (10.8)	< 0.001^1^
**Ethnicity**						< 0.001^2^
non-Sami	2462 (79.5%)	1319 (79.4%)	349 (68.4%)	920 (73.6%)	5050 (77.5%)	
Sami	633 (20.5%)	343 (20.6%)	161 (31.6%)	330 (26.4%)	1467 (22.5%)	
**Smoking**						< 0.001^2^
Yes, currently	1063 (34.3%)	588 (35.4%)	120 (23.5%)	277 (22.2%)	2048 (31.4%)	
Yes, previously	948 (30.6%)	481 (28.9%)	192 (37.6%)	441 (35.3%)	2062 (31.6%)	
Never	1084 (35.0%)	593 (35.7%)	198 (38.8%)	532 (42.6%)	2407 (36.9%)	
**Died during follow-up**	154 (5.0%)	230 (13.8%)	25 (4.9%)	187 (15.0%)	596 (9.1%)	< 0.001^2^
**Cause of death**						< 0.001^2^
Malignant tumor	83 (53.9%)	63 (27.4%)	12 (48.0%)	60 (32.1%)	218 (36.6%)	
CVD	16 (10.4%)	73 (31.7%)	5 (20.0%)	58 (31.0%)	152 (25.5%)	
Respiratory	19 (12.3%)	25 (10.9%)	3 (12.0%)	15 (8.0%)	62 (10.4%)	
Other	33 (21.4%)	67 (29.1%)	4 (16.0%)	51 (27.3%)	155 (26.0%)	
Unknown	3 (1.9%)	2 (0.9%)	1 (4.0%)	3 (1.6%)	9 (1.5%)	
**Alcohol consumption**						< 0.001^2^
Weekly	822 (26.6%)	296 (17.8%)	89 (17.5%)	132 (10.6%)	1339 (20.5%)	
Less than weekly	1881 (60.8%)	958 (57.6%)	312 (61.2%)	741 (59.3%)	3892 (59.7%)	
Never/not last year	392 (12.7%)	408 (24.5%)	109 (21.4%)	377 (30.2%)	1286 (19.7%)	
**Leisure-time physical activity**						< 0.001^2^
Sedentary	594 (19.2%)	394 (23.7%)	140 (27.5%)	397 (31.8%)	1525 (23.4%)	
Light	2082 (67.3%)	1100 (66.2%)	324 (63.5%)	751 (60.1%)	4257 (65.3%)	
Moderate-hard	419 (13.5%)	168 (10.1%)	46 (9.0%)	102 (8.2%)	735 (11.3%)	
**Education (years)**	12.6 (3.9)	10.6 (3.7)	11.6 (4.1)	10.5 (3.9)	11.6 (4.0)	< 0.001^1^
**General obesity**	0 (0.0%)	0 (0.0%)	510 (100.0%)	1250 (100.0%)	1760 (27.0%)	
**Metabolic syndrome**	0 (0.0%)	948 (57.0%)	0 (0.0%)	990 (79.2%)	1938 (29.7%)	< 0.001^2^
**Hypertension**	802 (25.9%)	1173 (70.6%)	176 (34.5%)	1023 (81.8%)	3174 (48.7%)	< 0.001^2^
**Increased waist circumference**	1274 (41.2%)	1267 (76.2%)	503 (98.6%)	1244 (99.5%)	4288 (65.8%)	< 0.001^2^
**Low HDL cholesterol**	542 (17.5%)	768 (46.2%)	102 (20.0%)	768 (61.4%)	2180 (33.5%)	< 0.001^2^
**Elevated triglycerides**	308 (10.0%)	810 (48.7%)	59 (11.6%)	792 (63.4%)	1969 (30.2%)	< 0.001^2^
**Hyperglycemia**	30 (1.0%)	157 (9.4%)	2 (0.4%)	194 (15.5%)	383 (5.9%)	< 0.001^2^
**Stroke**	0 (0.0%)	68 (4.5%)	0 (0.0%)	37 (3.2%)	105 (1.7%)	< 0.001^2^
Missing data	3	166	2	83	254	
**Angina pectoris**	0 (0.0%)	146 (9.8%)	0 (0.0%)	134 (11.4%)	280 (4.5%)	< 0.001^2^
Missing data	3	167	2	73	245	
**Myocardial infarction**	0 (0.0%)	58 (3.9%)	0 (0.0%)	36 (3.1%)	94 (1.5%)	< 0.001^2^
Missing data	3	165	2	80	250	
**Diabetes**	0 (0.0%)	101 (6.7%)	0 (0.0%)	133 (11.3%)	234 (3.7%)	< 0.001^2^
Missing data	3	163	2	74	242	
**Blood pressure-lowering drug**	0 (0.0%)	713 (43.8%)	0 (0.0%)	629 (50.9%)	1342 (20.8%)	< 0.001^2^
Missing data	3	36	2	14	55	
**Cholesterol-lowering drug**	0 (0.0%)	460 (29.0%)	0 (0.0%)	303 (25.5%)	763 (12.0%)	< 0.001^2^
Missing data	3	75	2	60	140	
**Glucose-lowering drug**	0 (0.0%)	96 (6.3%)	0 (0.0%)	108 (9.3%)	204 (3.2%)	< 0.001^2^
Missing data	3	136	2	93	234	

Continuous variables are reported as mean (standard deviation) and categorical variables are given as frequency (percent). In the final sample, missing data existed only in pre-existing disease and drug variables; in categorisation of metabolic health status, missing was assumed “no”, but frequencies of missing are shown in this table. It is evident that most people with missing nevertheless was categorised in an unhealthy group

HDL = high-density lipoprotein, CVD = cardiovascular disease

^1^One way analysis of variance

^2^Pearson’s χ^2^ test

### Statistical analysis

Sample characteristics were described in strata of sex and metabolic–obesity phenotype and reported as mean (SD) and frequency (percentage) as appropriate. One-way analysis of variance and Pearson’s χ^2^ test were used to compare characteristics across the phenotypes. We calculated age-standardised mortality rates using the direct method and the 2013 European standard population.

In separate models for each pair of outcome and exposure, we modelled the relationships between all-cause mortality and CVD mortality (outcomes) and MetS, general obesity phenotypes and abdominal obesity phenotypes (exposures) using Cox proportional hazard regression. We tested interactions between exposures and sex, and between exposures and ethnicity, and compared models with and without interaction terms using the likelihood ratio test. Interaction was considered present if *p* < 0.05. There were no significant interactions with ethnicity, but we found evidence of interactions between sex and general (*p* = 0.02) and abdominal (*p* = 0.05) obesity phenotypes for CVD mortality. Therefore, all models were stratified by sex. Attained age was set as the time-scale as recommended in observational studies [25], hence, all models were inherently and non-parametrically controlled for age (model 1). Further adjustments were made for smoking (model 2), plus leisure-time physical activity, education and alcohol consumption (model 3). Sami ethnicity is primarily regarded a sociocultural category in this cohort, and neither interacted with nor affected the beta coefficient for the exposures in the models, and was therefore not included in the models. The proportional hazard assumption was evaluated using Schoenfeld residuals. In models with all-cause mortality, non-proportional hazards for smoking status were handled by allowing separate baseline hazards for subgroups of the data, i.e. stratified Cox models. We reported adjusted hazard ratios (HR) with 95% confidence intervals (CI) for each pair of outcome and exposure.

Next, in separate models, we fitted BMI, waist circumference and ABSI as continuous variables using restricted cubic splines against all-cause and CVD mortality, respectively, while adjusting for the same covariates as in model 3 above, in addition to metabolic health. Fitting three knots provided the lowest Akaike information criterion and were thus sufficient, as recommended by Harrell [26]. We assessed non-linearity by testing models with the linear term against models with both linear and a cubic spline term using likelihood ratio test. Non-linearity was considered present if *p* < 0.05. We also assessed interaction between metabolic health status and BMI/waist circumference/ABSI using likelihood ratio tests. If there was a significant interaction, we kept the interaction term in the model; if there was no interaction, metabolic health status was kept in the model as a covariate. Adjusted HR (95% CI) of all-cause and CVD mortality, respectively, were plotted against BMI, waist circumference and ABSI, respectively, with separate curves for metabolically healthy and unhealthy, using the sex-specific sample median of BMI, waist circumference or ABSI as reference values. In models with a significant interaction, metabolically healthy with the sex-specific sample median of BMI, waist circumference or ABSI were used as reference.

We used *R* version 3.6.2 for Windows for statistical computing [27]. Code and output is found in the Additional file 1

### Sensitivity analysis

We excluded (1) ever-smokers and (2) participants with pre-existing diseases (or prescribed drugs for cardiometabolic disease) in sensitivity analyses. Furthermore, we analysed data with more conservative cut-offs for MetS-components: waist circumference (≥ 88/102 cm in women/men), random triglycerides (≥ 2.1 mmol/L), and random glucose (≥ 11.1 mmol/L). We also repeated the analyses in the full sample, adjusting for sex. Finally, we used multiple imputation to address missing data on at least one variable for 2030 participants (13.7%). The variables with the largest proportion of missing data were found for leisure-time physical activity (n = 1322, 8.9%) and education (n = 881, 5.9%). Characteristics differed between participants with complete and missing data (Additional file 1: Table S1). The mechanism for missing information was assumed to be missing-at-random [28]. We used a rich set of relevant variables, performed 20 imputations, and pooled the data according to Rubin’s rules using the ‘mice’ package in *R* [29]. Because metabolic health is a known mediator of the relationship between obesity and mortality, we also ran the analyses of continuous BMI/waist circumference/ABSI vs mortality without adjusting for metabolic health.

## Results

After median follow-up of 15.3 years in 6517 women and 15.2 years in 6298 men (12,815 in total), 596 (9.1%) and 938 (14.9%) had died, respectively. In both women and men, the prevalence of MetS was 29.7%. Proportions categorised as metabolically unhealthy (defined as either having MetS, pre-existing disease or prescribed drugs) were 44.7% in women and 47.0% in men. Proportions having general obesity were 27.0% in women and 23.5% in men, and proportions having abdominal obesity were 39.0% in women and 21.1% in men.

Tables 1 and 2 describe the prevalence of the four general obesity phenotypes and the distributions of characteristics across the phenotypes in women and men, respectively. Compared to the other groups, men and women with MHO were relatively young, with a higher proportion of people with Sami ethnicity, a lower proportion of current smokers, and a higher proportion of people who reported being sedentary in their leisure-time (but lower than in people with MUO). Additional file 1: Tables S2 and S3 describe the distribution and characteristics of the four abdominal obesity phenotypes. Patterns of characteristics were generally similar to those reported for general obesity phenotypes.

**Table.2:** Sample characteristics in mean (standard deviation) or frequency (percent) according to general obesity phenotypes in 6298 men in the SAMINOR 1 Survey (2003–2004)

	Metabolically healthy non-obesity (N = 2972, 47.2%)	Metabolically unhealthy non-obesity (N = 1843, 29.2%)	Metabolically healthy obesity (N = 363, 5.8%)	Metabolically unhealthy obesity (N = 1120, 17.8%)	Total (N = 6298)	p-value
**Age (years)**	51.4 (9.9)	57.8 (10.8)	51.3 (10.1)	55.4 (10.3)	54.0 (10.6)	< 0.001^1^
**Ethnicity**						0.002^2^
non-Sami	2264 (76.2%)	1452 (78.8%)	253 (69.7%)	865 (77.2%)	4834 (76.8%)	
Sami	708 (23.8%)	391 (21.2%)	110 (30.3%)	255 (22.8%)	1464 (23.2%)	
**Smoking**						< 0.001^2^
Yes, currently	1060 (35.7%)	549 (29.8%)	86 (23.7%)	260 (23.2%)	1955 (31.0%)	
Yes, previously	982 (33.0%)	830 (45.0%)	158 (43.5%)	571 (51.0%)	2541 (40.3%)	
Never	930 (31.3%)	464 (25.2%)	119 (32.8%)	289 (25.8%)	1802 (28.6%)	
**Died during follow-up**	297 (10.0%)	402 (21.8%)	39 (10.7%)	200 (17.9%)	938 (14.9%)	< 0.001^2^
**Cause of death**						< 0.001^2^
Malignant tumor	124 (41.8%)	123 (30.6%)	12 (30.8%)	63 (31.5%)	322 (34.3%)	
CVD	56 (18.9%)	135 (33.6%)	18 (46.2%)	75 (37.5%)	284 (30.3%)	
Respiratory	38 (12.8%)	47 (11.7%)	5 (12.8%)	14 (7.0%)	104 (11.1%)	
Other	75 (25.3%)	91 (22.6%)	3 (7.7%)	41 (20.5%)	210 (22.4%)	
Unknown	4 (1.3%)	6 (1.5%)	1 (2.6%)	7 (3.5%)	18 (1.9%)	
**Alcohol consumption**						< 0.001^2^
Weekly	1046 (35.2%)	545 (29.6%)	117 (32.2%)	315 (28.1%)	2023 (32.1%)	
Less than weekly	1691 (56.9%)	1057 (57.4%)	213 (58.7%)	683 (61.0%)	3644 (57.9%)	
Never/not last year	235 (7.9%)	241 (13.1%)	33 (9.1%)	122 (10.9%)	631 (10.0%)	
**Leisure-time physical activity**						< 0.001^2^
Sedentary	602 (20.3%)	417 (22.6%)	93 (25.6%)	339 (30.3%)	1451 (23.0%)	
Light	1571 (52.9%)	1088 (59.0%)	200 (55.1%)	616 (55.0%)	3475 (55.2%)	
Moderate-hard	799 (26.9%)	338 (18.3%)	70 (19.3%)	165 (14.7%)	1372 (21.8%)	
**Education (years)**	11.7 (3.8)	10.6 (3.7)	11.2 (3.4)	10.8 (3.7)	11.2 (3.8)	< 0.001^1^
**General obesity**	0 (0.0%)	0 (0.0%)	363 (100.0%)	1120 (100.0%)	1483 (23.5%)	
**Metabolic syndrome**	0 (0.0%)	970 (52.6%)	0 (0.0%)	900 (80.4%)	1870 (29.7%)	< 0.001^2^
**Hypertension**	1271 (42.8%)	1493 (81.0%)	164 (45.2%)	972 (86.8%)	3900 (61.9%)	< 0.001^2^
**Increased waist circumference**	636 (21.4%)	1031 (55.9%)	331 (91.2%)	1097 (97.9%)	3095 (49.1%)	< 0.001^2^
**Low HDL cholesterol**	258 (8.7%)	592 (32.1%)	22 (6.1%)	488 (43.6%)	1360 (21.6%)	< 0.001^2^
**Elevated triglycerides**	825 (27.8%)	1040 (56.4%)	93 (25.6%)	815 (72.8%)	2773 (44.0%)	< 0.001^2^
**Hyperglycemia**	44 (1.5%)	230 (12.5%)	3 (0.8%)	163 (14.6%)	440 (7.0%)	< 0.001^2^
**Stroke**	0 (0.0%)	100 (5.9%)	0 (0.0%)	51 (4.8%)	151 (2.5%)	< 0.001^2^
Missing data	6	145	0	52	203	
**Angina pectoris**	0 (0.0%)	318 (18.6%)	0 (0.0%)	138 (12.9%)	456 (7.5%)	< 0.001^2^
Missing data	6	137	0	48	191	
**Myocardial infarction**	0 (0.0%)	236 (13.7%)	0 (0.0%)	110 (10.2%)	346 (5.7%)	< 0.001^2^
Missing data	6	124	0	45	175	
**Diabetes**	0 (0.0%)	135 (7.9%)	0 (0.0%)	85 (7.9%)	220 (3.6%)	< 0.001^2^
Missing data	6	134	0	45	185	
**Blood pressure-lowering drug**	0 (0.0%)	837 (46.4%)	0 (0.0%)	504 (45.4%)	1341 (21.5%)	< 0.001^2^
Missing data	6	38	0	10	54	
**Cholesterol-lowering drug**	0 (0.0%)	630 (35.6%)	0 (0.0%)	320 (29.5%)	950 (15.4%)	< 0.001^2^
Missing data	6	74	0	35	115	
**Glucose-lowering drug**	0 (0.0%)	131 (7.7%)	0 (0.0%)	66 (6.3%)	197 (3.2%)	< 0.001^2^
Missing data	6	141	0	68	215	

Continuous variables are reported as mean (standard deviation) and categorical variables are given as frequency (percent). In the final sample, missing data existed only in pre-existing disease and drug variables; in categorisation of metabolic health status, missing was assumed “no”, but frequencies of missing are shown in this table. It is evident that most people with missing nevertheless was categorised in an unhealthy group

HDL = high-density lipoprotein, CVD = cardiovascular disease

^1^One way analysis of variance

^2^Pearson’s χ^2^ test

**Table.3:** All-cause and CVD mortality according to MetS, general and abdominal obesity phenotypes: Hazard ratios (HR) and 95% confidence intervals (CI) from Cox proportional hazards models of 6517 women in the SAMINOR 1 Survey (2003–2004)

				Model 1		Model 2		Model 3	
	Cases	Person-years	IR	HR	95% CI	HR	95% CI	HR	95% CI
**Outcome: All-cause mortality**									
**Metabolic syndrome**									
No	343	68,588.7	5.0	Ref		Ref		Ref	
Yes	253	28,604.7	8.8	1.14	0.97–1.35	1.15	0.97–1.35	1.11	0.94–1.31
**General obesity phenotypes**									
Metabolically healthy non-obese	154	46,629.4	3.3	Ref		Ref		Ref	
Metabolically unhealthy non-obese	230	24,487.6	9.4	1.13	0.92–1.40	1.14	0.92–1.41	1.11	0.90–1.38
Metabolically healthy obese	25	7753.5	3.2	0.64	0.42–0.97	0.68	0.44–1.04	0.63	0.41–0.97
Metabolically unhealthy obese	187	18,322.8	10.2	1.17	0.94–1.46	1.27	1.02–1.59	1.17	0.93–1.47
**Abdominal obesity phenotypes**									
Metabolically healthy non-abdominally obese	119	39,259.1	3.0	Ref		Ref		Ref	
Metabolically unhealthy non-abdominally obese	170	20,308.6	8.4	1.12	0.88–1.43	1.14	0.89–1.45	1.12	0.88–1.43
Metabolically healthy abdominally obese	42	12,571.2	3.3	0.71	0.50–1.01	0.75	0.53–1.07	0.71	0.50–1.02
Metabolically unhealthy abdominally obese	265	25,054.5	10.6	1.23	0.99–1.55	1.31	1.04–1.64	1.22	0.97–1.54
**Outcome: CVD mortality**									
**Metabolic syndrome**									
No	73	68,588.7	1.1	Ref		Ref		Ref	
Yes	79	28,604.7	2.8	1.55	1.12–2.13	1.53	1.11–2.11	1.46	1.06–2.02
**General obesity phenotypes**									
Metabolically healthy non-obese	16	46,629.4	0.3	Ref		Ref		Ref	
Metabolically unhealthy non-obese	73	24,487.6	3.0	2.86	1.65–4.95	2.88	1.66–4.99	2.77	1.59–4.80
Metabolically healthy obese	5	7753.5	0.6	1.08	0.40–2.96	1.12	0.41–3.07	1.05	0.38–2.88
Metabolically unhealthy obese	58	18,322.8	3.2	2.81	1.60–4.94	2.93	1.66–5.15	2.65	1.49–4.72
**Abdominal obesity phenotypes**									
Metabolically healthy non-abdominally obese	16	39,259.1	0.4	Ref		Ref		Ref	
Metabolically unhealthy non-abdominally obese	48	20,308.6	2.4	1.90	1.07–3.38	1.93	1.09–3.43	1.86	1.05–3.32
Metabolically healthy abdominally obese	5	12,571.2	0.4	0.55	0.20–1.50	0.57	0.21–1.56	0.54	0.20–1.47
Metabolically unhealthy abdominally obese	83	25,054.5	3.3	2.25	1.30–3.88	2.31	1.34–3.99	2.11	1.21–3.69

Model 1 is the crude model (all models inherently adjusted for age by using attained age as the time-scale). Model 2 was additionally adjusted for smoking, and model 3 was additionally adjusted for leisure-time physical activity, education and alcohol consumption (model 3). We applied stratified Cox models with separate baseline hazards for subgroups of smoking status to satisfy the proportional hazard assumption in all-cause mortality models

IR = crude incidence rate per 1000 person-years, HR = hazard ratio, CI = confidence interval

The proportion of deaths during follow-up were comparable in people with MHO and people with MHNO, but they differed in the distribution of causes of death (Tables 1 and 2). In general, the proportion of death from CVD was lowest in the MHNO group.

**Fig. 2:**
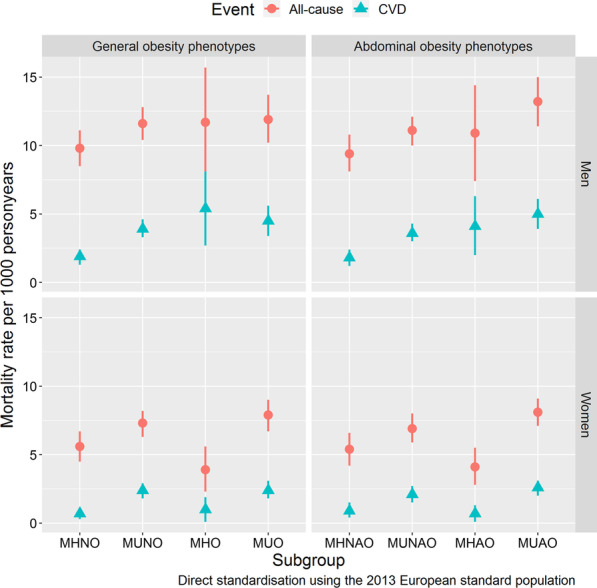
Age-standardised mortality rates per 1000 person-years with 95% CI for all-cause and CVD mortality given by general and abdominal obesity phenotypes. MHNO = metabolically healthy non-obesity, MUNO = metabolically unhealthy non-obesity, MHO = metabolically healthy obesity, MUO = metabolically unhealthy obesity, MHNAO = metabolically healthy non-abdominal obesity, MUNAO = metabolically unhealthy non-abdominal obesity, MHAO = metabolically healthy abdominal obesity, MUAO = metabolically unhealthy abdominal obesity

Figure 2 shows that the lowest mean mortality rates in men occurred in the MHNO and MHNAO groups, whereas in women, the metabolically healthy phenotypes regardless of obesity status had the lowest mortality rates.

Tables 3 and 4 show the hazard ratios (HR) from Cox proportional hazards models for all-cause mortality and CVD mortality in women and men, respectively. Men and women with MetS had an approximately 50% higher 15-year risk of CVD mortality than those without MetS. The 15-year mortality in the subgroups with MHO and MHAO compared to the respective metabolically healthy non-obese groups differed markedly between the sexes, particularly for CVD mortality, with significant interactions with sex differences in the beta coefficient for MHO and MHAO primarily. We found that obesity, regardless of metabolic health, markedly increased CVD mortality in men, but there was no association in women. In the metabolically healthy, all-cause mortality was reduced in obese women (general and abdominal, respectively) compared to non-obese women. In both sexes, the mortality associated with metabolically unhealthy obesity phenotypes (MUNO, MUNAO, MUO, MUAO) were higher for CVD-specific death than for all-cause mortality.

**Table.4:** All-cause and CVD mortality according to MetS, general and abdominal obesity phenotypes: Hazard ratios (HR) and 95% confidence intervals (CI) from Cox proportional hazards models of 6298 men in SAMINOR 1 (2003–2004)

				Model 1		Model 2		Model 3	
	Cases	Person-years	IR	HR	95% CI	HR	95% CI	HR	95% CI
Outcome: All-cause mortality									
**Metabolic syndrome**									
No	627	65,040.4	9.6	Ref		Ref		Ref	
Yes	311	27,124.8	11.5	1.06	0.93–1.22	1.11	0.97–1.28	1.10	0.96–1.26
**General obesity phenotypes**									
Metabolically healthy non-obese	297	44,234.7	6.7	Ref		Ref		Ref	
Metabolically unhealthy non-obese	402	26,321.0	15.3	1.12	0.96–1.31	1.18	1.01–1.38	1.16	0.99–1.35
Metabolically healthy obese	39	5381.8	7.2	1.13	0.81–1.57	1.28	0.91–1.79	1.25	0.89–1.75
Metabolically unhealthy obese	200	16,227.8	12.3	1.22	1.02–1.46	1.38	1.14–1.65	1.33	1.11–1.61
**Abdominal obesity phenotypes**									
Metabolically healthy non-abdominally obese	241	38,178.8	6.3	Ref		Ref		Ref	
Metabolically unhealthy non-abdominally obese	430	34,896.0	12.3	1.13	0.97–1.33	1.20	1.02–1.41	1.18	1.00–1.38
Metabolically healthy abdominally obese	40	4344.3	9.2	1.12	0.80–1.57	1.23	0.88–1.73	1.20	0.86–1.69
Metabolically unhealthy abdominally obese	227	14,746.1	15.4	1.39	1.16–1.67	1.53	1.27–1.84	1.49	1.23–1.79
**Outcome: CVD mortality**									
**Metabolic syndrome**									
No	170	65,040.4	2.6	Ref		Ref		Ref	
Yes	114	27,124.8	4.2	1.43	1.13–1.82	1.53	1.20–1.94	1.51	1.18–1.91
**General obesity phenotypes**									
Metabolically healthy non-obese	56	44,234.7	1.3	Ref		Ref		Ref	
Metabolically unhealthy non-obese	135	26,321.0	5.1	1.95	1.42–2.68	2.11	1.54–2.90	2.08	1.51–2.86
Metabolically healthy obese	18	5381.8	3.3	2.68	1.57–4.56	3.03	1.77–5.19	2.92	1.71–5.01
Metabolically unhealthy obese	75	16,227.8	4.6	2.40	1.69–3.40	2.83	1.98–4.03	2.72	1.90–3.89
**Abdominal obesity phenotypes**									
Metabolically healthy non-abdominally obese	47	38,178.8	1.2	Ref		Ref		Ref	
Metabolically unhealthy non-abdominally obese	137	34,896.0	3.9	1.81	1.30–2.54	1.98	1.41–2.76	1.94	1.38–2.72
Metabolically healthy abdominally obese	15	4344.3	3.5	2.07	1.15–3.70	2.28	1.27–4.09	2.18	1.21–3.92
Metabolically unhealthy abdominally obese	85	14,746.1	5.8	2.61	1.82–3.74	3.00	2.08–4.32	2.89	2.00–4.17

Model 1 is the crude model (all models inherently adjusted for age by using attained age as the time-scale). Model 2 was additionally adjusted for smoking, and model 3 was additionally adjusted for leisure-time physical activity, education and alcohol consumption (model 3). We applied stratified Cox models with separate baseline hazards for subgroups of smoking status to satisfy the proportional hazard assumption in all-cause mortality models

IR = crude incidence rate per 1000 person-years, HR = hazard ratio, CI = confidence interval

**Fig. 3:**
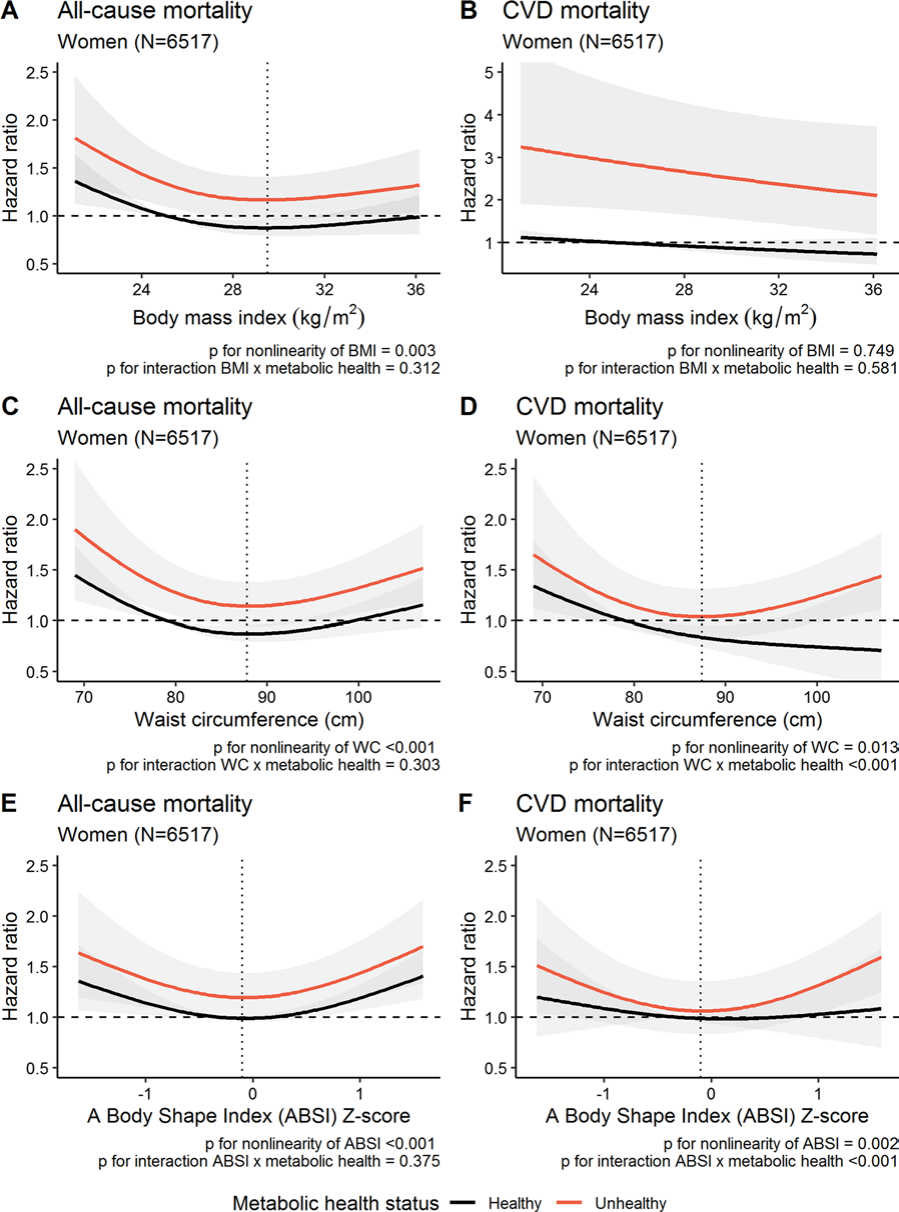
The functional relationships between mortality (all-cause and CVD) and continuous obesity measures (BMI, waist circumference and ABSI) with corresponding hazard ratios with 95% confidence bands in women. The reference of all curves were metabolically healthy women with a BMI of 26.7 kg/m^2^, a waist circumference of 79 cm or an ABSI Z-score of −0.32 (median values for metabolically healthy women). *p* Values originates from likelihood ratio tests comparing models with/without linear terms/interaction terms. The beta coefficient for metabolic health status was statistically significant in all models. Estimates are predicted for median values of confounders (smoking, leisure-time physical activity, education, alcohol consumption). All models were inherently adjusted for age by using attained age as the time-scale. The vertical, dotted lines represent the nadir of risk. In panel **D**, the nadir of risk of metabolically healthy and unhealthy differ due to a significant interaction (nadir lower in unhealthy than healthy). Note that panel **B** has different dimensions on the y-axis than the other panels. ABSI = a body shape index, BMI = body mass index, WC = waist circumference

**Fig. 4:**
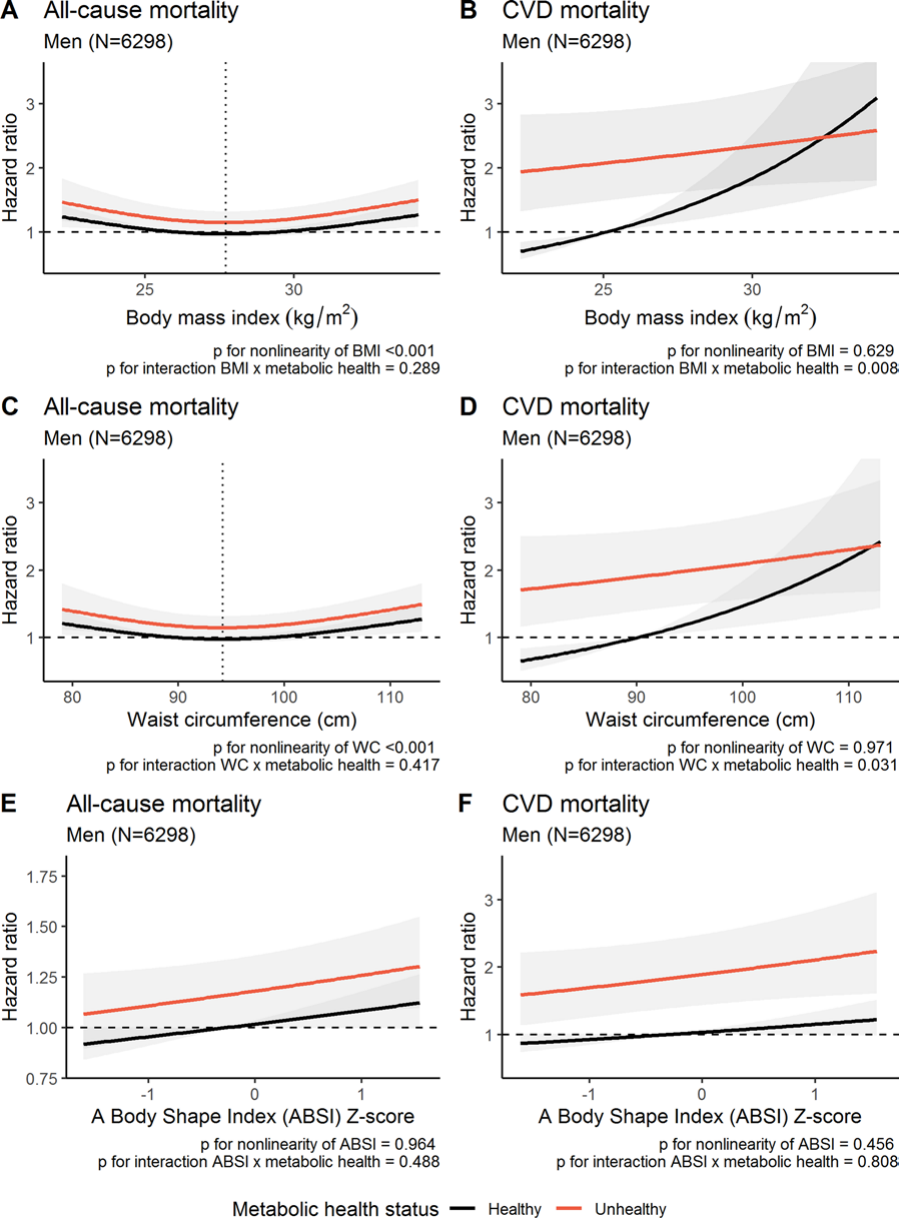
The functional relationships between mortality (all-cause and CVD) and continuous obesity measures (BMI, waist circumference and ABSI) with corresponding hazard ratios with 95% confidence bands in men. The reference of all curves were metabolically healthy men with a BMI of 27.2 kg/m^2^, a waist circumference of 90 cm or an ABSI Z-score of −0.28 (median values for metabolically healthy men). *P* values originates from likelihood ratio tests comparing models with/without linear terms/interaction terms. The beta coefficient for metabolic health status was statistically significant in all models. Estimates are predicted for median values of confounders (smoking, leisure-time physical activity, education, alcohol consumption). All models were inherently adjusted for age by using attained age as the time-scale. The vertical, dotted lines represent the nadir of risk. ABSI = a body shape index, BMI = body mass index, WC = waist circumference

Figures 3 and 4 (panels A and C) show curvilinear relationships between all-cause mortality and BMI (panel A) and waist circumference (panel C) in women and men, respectively. Figures 3 and 4 (panels E) show curvilinear and linear relationships between all-cause mortality and ABSI in women and men, respectively. Figures 3 and 4 (panels B, D and F) show marked sex-differences in the relationships with CVD mortality for BMI (panel B), waist circumference (panel D) and ABSI (panel F). Interactions were present between metabolic health status and obesity measures in CVD models (except in panel 3B and 4F). In men, all obesity measures had positive, strong associations with CVD mortality. We found stronger associations (steeper slopes) in metabolically healthy than unhealthy groups in models with BMI and waist circumference, but not in models with ABSI. In women, BMI had negative associations with CVD mortality. The association between waist circumference or ABSI and CVD mortality differed by metabolic health status.

### Sensitivity analysis

Additional file 1: Tables S4, S5 and S6 show the results of the sensitivity analyses. In never-smokers, most associations between general and abdominal obesity phenotypes and mortality were stronger than those observed in the whole cohort, but several estimates included 1.0 in the CI. Contrary, in participants without pre-existing disease or prescribed drugs, most estimates were strongly attenuated and not statistically significant (except men with MHO and MHAO) compared to those observed in the whole cohort. Using more conservative cut-offs for MetS resulted in increased estimates, and the apparent protective effect of MHO and MHAO in women was attenuated towards the null and was no longer statistically significant. In sex-adjusted analyses, HR (95%) for all-cause mortality compared to the reference groups were 0.92 (0.71–1.20) for MHO and 0.92 (0.72–1.17) for MHAO, respectively. Analysis of multiply imputed data gave similar results compared to the complete case analysis. Additional file 1: Figs. S1 and S2 of “unadjusted” obesity vs mortality models show overall patterns similar with the primary analyses. An exception was seen for models with CVD mortality in women (Figure S1), which showed no association with BMI (panel B) or waist circumference (panel D), but a curvilinear association with ABSI (panel F) indicating significantly higher mortality at higher ends of the scale.

## Discussion

We followed almost 13,000 adults for 15 years and found that metabolically unhealthy status was associated with a higher CVD mortality than metabolically healthy status irrespective of obesity status. We found curvilinear associations between BMI (women and men), waist circumference (women and men) or ABSI (women) and all-cause mortality regardless of metabolic health status. However, in men, the relationship between ABSI and all-cause mortality was linear. Corresponding relationships between these three continuous obesity measures and CVD mortality differed by both sex and metabolic health status. Ethnicity had no impact on the results.

To our knowledge, this study is the first to examine the relationship between continuous measures of BMI, waist circumference or ABSI and mortality according to metabolic health status. A recent study of a Japanese population by Izumida et al. examined the relationships between four categories of BMI and 18-year mortality according to MetS status [30]. The relationship between BMI categories and all-cause and CVD mortality were J-shaped in metabolically unhealthy people, whereas no associations were found in metabolically healthy people. In contrast, we show that the relationships between BMI and CVD mortality in a Norwegian population differ by sex: with no or negative association in women and positive association in men. A meta-analysis of 21 prospective studies showed that compared to the MHNO group, the HR for CVD in women with MHO were lower than those in men with MHO (HR 1.71 vs 2.15, respectively) [31]. However, the meta-analysis included few sex-stratified studies. In a recent Iranian study, neither women nor men with persistent MHO status had increased HR for CVD incidence compared to the non-obese comparison group [32]. However, among women and men who transitioned from MHO to MUO, only men had an increased HR compared to the non-obese comparison group [32]. In the study by Izumida et al., the authors adjusted for sex, whereas we found an interaction, but only regarding CVD mortality. The association between BMI/waist circumference and all-cause mortality was U-shaped in both sexes. Although the HR of MHO for all-cause mortality differed by sex (HR of 0.63 in women and 1.25 in men), there was no evidence of statistically significant effect modification. In sensitivity analyses, the (sex-adjusted) HR (95% CI) of MHO was 0.92 (0.71–1.21).

The amount of visceral adipose tissue may differ between people with the same value of BMI or even waist circumference, and men typically have more visceral adipose tissue than women [13]. This may have contributed to the sex-differences in associations between obesity measures and CVD mortality in women and men. A recent UK Biobank study including nearly 300,000 men and women without CVD at baseline showed that BMI had J-shaped associations with CVD events and mortality in both sexes [33]. In men, the association with CVD events was linear when restricted to non-smokers. Residual confounding when adjusting for crude smoking categories has been pointed out as a potential cause of obesity paradoxes [34]. We also show that when the analyses were restricted to non-smokers, most estimates increased, and women with MHO had a HR of approximately 1.50 for CVD mortality, albeit non-statistically significant due to low power. Importantly, in the UK Biobank study, all measures of central obesity, including waist circumference, and fat mass were positively associated with CVD mortality in both sexes [33].

A high ABSI seems to be a more consistent predictor of mortality in both women and men compared to a high BMI or waist circumference irrespective of metabolic health status; however, we have not formally compared the models. Studies in a US and four European (Sweden, Finland, Turkey and UK) cohorts have shown that where BMI or waist circumference tend to show curvilinear relationships with mortality, a progressively increasing ABSI corresponds to an increasing mortality [17, 35]. As opposed to BMI and waist circumference, ABSI was linearly and positively associated with both all-cause and CVD mortality in men. This pattern for ABSI was not found in women, perhaps owing to the weak, but existing correlation with BMI (0.17 in women vs 0.08 in men). Ideally, the correlation between ABSI and BMI should be null [17], but due to differences in distributions of height, weight and waist circumference between the participants in the NHANES and the SAMINOR Study, the formula is not a perfect fit in the latter. Recently, ABSI was derived specifically for the UK Biobank population [36], and in the future deriving population-specific formulae may avoid bias from correlations with BMI.

In models not controlling for metabolic health, we found linear (men) and U-shaped (women) associations between ABSI and both all-cause and CVD mortality (Additional file 1: Figs. S1 and S2). In women, ABSI scores above the mean were strongly associated with mortality. At the lower end of ABSI, CIs were wide. In a recent study using a large European cohort, the ABSI—mortality relationship also differed by sex [37]. In women, the relationship was J-shaped, with positive associations only in the higher quintiles, whereas ABSI was positively associated with mortality in all quintiles in men. Our results show some similarity to these findings. The aforementioned study showed that people with a high ABSI had approximately 30% higher mortality compared to people with low ABSI, irrespective of BMI category [37]. This suggests that ABSI reflects an altered, detrimental body shape that is not reflected in BMI. A small study found that ABSI and BMI were negatively and positively, respectively, associated with fat free mass, or lean mass, indicating that a high ABSI is a good marker of sarcopenic obesity [38]. In future studies, it may be interesting to replace BMI with ABSI in defining categorical obesity phenotypes, i.e., to define a MHO phenotype from body shape.

Collider bias has been suggested to explain the “obesity paradox”: obesity increases mortality and causes cardiometabolic disease, but within strata of cardiometabolic disease, obesity is not associated with mortality or even appears protective in some studies [39, 40], as is seen in models with BMI and waist circumference for women in this study. The collider bias is a type of selection bias, that can be introduced through restriction, regression adjustment or stratification on a variable (in this case cardiometabolic status) that is both affected by the exposure (obesity) and share common causes (e.g. genes) with the outcome (death). However, the magnitude and direction of the bias may be difficult to predict, and some suggest it only a partial explanation of the obesity paradox [41].

Izumida et al. defined metabolically healthy as having no MetS components, compared to our definition of two or fewer components. Hence, metabolically healthy people in our study may have been in a transition phase towards full MetS and converted to metabolically unhealthy during the study period. Approximately 50% of people with MHO transition to MUO [4]. A study with six repeated measures during 30 years of follow-up showed that duration with MHO was longer in women than in men. Women transitioned back and forth between a healthy and an unhealthy metabolic status while maintaining their obesity status, whereas men with MHO tended to just transition once from a healthy to an unhealthy metabolic status [42]. Nevertheless, in a large U.S. cohort of women (N≈90,000), both those with MHO at baseline and those with persistent MHO status over a period of 24 years were at increased risk of CVD compared with the MHNO [43]. Hence, even if women spend a longer time in the MHO state before transitioning to MUO than men, MHO may not be a benign state in a perspective of several decades.

Furthermore, in a study with repeated measures, people with MHO had higher all-cause mortality only when compared to people with stable MHNO status identified during several assessments, and not in comparison to the larger group that were MHNO at baseline [44]. This serves as a reminder that exposure status in the reference group can change over time and a single measurement at baseline may give biased results. The implications for this study is that the strength of associations may have been under-estimated.

In summary, collider bias, residual confounding by smoking and misclassification may have distorted some of the relationships between obesity and mortality that we observed. The pathways linking obesity, metabolic health and mortality is complex and dynamic, making it a challenge to study using only data measured at a single point in time. Although obesity is heterogeneous in presentation, it is unlikely a healthy state over time, as is evident particularly for the men in our study.

### Strengths and limitations

Strengths of the study include the population-based nature of the study, the long follow-up time and standardised measurements of clinical and biochemical variables by trained personnel. Linkage to the high quality Norwegian Cause of Death Registry enabled virtually complete follow-up of total and CVD deaths. We included important confounders, such as physical activity, smoking, alcohol and education. However, we did not have information on occupational physical activity, which may comprise a large part of the total physical activity level throughout the day. Therefore, some residual confounding from physical activity may be present. Further limitations include non-fasting blood samples, and a modest participation rate that may have resulted in ‘healthy participation’ bias. There are no valid cut-offs for random glucose regarding prediabetes or impaired glucose tolerance. Non-fasting triglycerides reflect increases over fasting values by a maximum of 0.3 mmol/L [45]. Inclusion of inflammation markers (e.g. C-reactive protein) and information on non-alcoholic fatty liver disease may have enabled us to categorise more precisely into metabolically healthy vs unhealthy.

## Conclusion

Metabolically unhealthy people have increased risks of 15-year all-cause and CVD mortality irrespective of obesity status compared to people who were metabolically healthy at baseline. Associations between BMI, waist circumference or ABSI and CVD mortality differed between the sexes, with strong, positive associations in both metabolically healthy and unhealthy men. The relationship between metabolic risk factors and adipose tissue is dynamic and continuous; therefore, efforts should continue to be made to reduce obesity and metabolic abnormalities across the population.

## Supplementary Information

**Additional file 1. Supplementary Table 1**. Descriptive characteristics among participants with complete case data and participants with one or more missing data in 14,845 participants in the SAMINOR 1 Survey (2003―2004).** Supplementary Table 2**. Sample characteristics in mean (standard deviation) or frequency (percent) according to abdominal obesity phenotypes in 6517 women in the SAMINOR Study (2003―2004).** Supplementary Table 3**. Sample characteristics in mean (standard deviation) or frequency (percent) according to abdominal obesity phenotypes in 6298 men in the SAMINOR Study (2003―2004).** Supplementary Table 4**. Sensitivity analyses. Hazard ratio (HR) and 95% confidence interval (CI) of metabolic syndrome (MetS), general and abdominal obesity phenotypes for all-cause mortality and CVD mortality in various samples of women in the SAMINOR 1 Survey (2003–2004).** Supplementary Table 5**. Sensitivity analyses. Hazard ratio (HR) and 95% confidence interval (CI) of metabolic syndrome (MetS), general and abdominal obesity phenotypes for all-cause mortality and CVD mortality in various samples of men in the SAMINOR 1 Survey (2003–2004).** Supplementary Table 6**. All-cause and CVD mortality according to MetS, general and abdominal obesity phenotypes: Hazard ratios (HR) and 95% confidence intervals (CI) from Cox proportional hazards models of 12,815 men and women in SAMINOR 1 (2003–2004).** Supplementary Figure 1**. The functional relationships between mortality (all-cause and CVD) and continuous obesity measures (body mass index, waist circumference and a body shape index) with corresponding hazard ratios with 95% confidence bands in women. The reference of all curves were women with a BMI of 26.7 kg/m2, a waist circumference of 79 cm and a body shape index Z-score of 0 (median values). P-values originates from likelihood ratio tests comparing models with/without linear terms terms. Estimates are predicted for medianvalues of confounders (smoking, leisure-time physical activity, education, alcohol consumption). All models were inherently adjusted for age by using attained age as the time-scale. The vertical, dotted lines represent the nadir of risk. ABSI = a body shape index, BMI = body mass index, WC = waist circumference.** Supplementary Figure 2**. The functional relationships between mortality (all-cause and CVD) and continuous obesity measures (body mass index, waist circumference and a body shape index) with corresponding hazardratios with 95% confidence bands in men. The reference of all curves were men with a BMI of 27.2, a waist circumference of 90 cm and a body shape index Z-score of 0 (median values). P-values originates from likelihood ratio tests comparing models with/without linear terms terms. Estimates are predicted for median values of confounders (smoking, leisure-time physical activity, education, alcohol consumption). All models were inherently adjusted for age by using attained age as the time-scale. The vertical, dotted lines represent the nadir of risk. ABSI = a body shape index, BMI = body mass index, WC = waist circumference.

## Data Availability

The datasets generated and/or analysed during the current study are not publicly available due to privacy regulations. Data from the SAMINOR Study may be made available upon reasonable request to the SAMINOR Project Board and with permission of the Regional Committee for Medical and Health Research Ethics.

## Abbreviations

MHNO: Metabolically healthy non-obesity
MUNO: Metabolically unhealthy non-obesity
MHO: Metabolically healthy obesity
MUO: Metabolically unhealthy obesity
MHNAO: Metabolically healthy non-abdominal obesity
MUNAO: Metabolically unhealthy non-abdominal obesity
MHAO: Metabolically healthy abdominal obesity
MUAO: Metabolically unhealthy abdominal obesity
MetS: Metabolic syndrome, CVD: cardiovascular disease
BMI: Body mass index
ABSI: A body shape index
HR: Hazard ratio
CI: Confidence interval
SD: Standard deviation
HDL: High density lipoprotein
