# Supplementary material for: Obesity measures, metabolic health and their association with 15-year all-cause and cardiovascular mortality in the SAMINOR 1 Survey: a population-based cohort study
Source: BMC Cardiovasc Disord. 2021 Oct 21;21:510. doi: 10.1186/s12872-021-02288-9 (PMC8529837; doi:10.1186/s12872-021-02288-9)
Supplement: Supplementary file 1 — Additional file 1. Supplementary Table 1. Descriptive characteristics among participants with complete case data and participants with one or more missing data in 14,845 participants in the SAMINOR 1 Survey (2003―2004). Supplementary Table 2. Sample characteristics in mean (standard deviation) or frequency (percent) according to abdominal obesity phenotypes in 6517 women in the SAMINOR Study (2003―2004). Supplementary Table 3. Sample characteristics in mean (standard deviation) or frequency (percent) according to abdominal obesity phenotypes in 6298 men in the SAMINOR Study (2003―2004). Supplementary Table 4. Sensitivity analyses. Hazard ratio (HR) and 95% confidence interval (CI) of metabolic syndrome (MetS), general and abdominal obesity phenotypes for all-cause mortality and CVD mortality in various samples of women in the SAMINOR 1 Survey (2003–2004). Supplementary Table 5. Sensitivity analyses. Hazard ratio (HR) and 95% confidence interval (CI) of metabolic syndrome (MetS), general and abdominal obesity phenotypes for all-cause mortality and CVD mortality in various samples of men in the SAMINOR 1 Survey (2003–2004). Supplementary Table 6. All-cause and CVD mortality according to MetS, general and abdominal obesity phenotypes: Hazard ratios (HR) and 95% confidence intervals (CI) from Cox proportional hazards models of 12,815 men and women in SAMINOR 1 (2003–2004). Supplementary Figure 1. The functional relationships between mortality (all-cause and CVD) and continuous obesity measures (body mass index, waist circumference and a body shape index) with corresponding hazard ratios with 95% confidence bands in women. The reference of all curves were women with a BMI of 26.7 kg/m2, a waist circumference of 79 cm and a body shape index Z-score of 0 (median values). P-values originates from likelihood ratio tests comparing models with/without linear terms terms. Estimates are predicted for medianvalues of confounders (smoking, leisure-time physical activity, education [file 12872_2021_2288_MOESM1_ESM.pdf]

Supplementary Table 1. Descriptive characteristics among participants with complete case data and participants with one or more missing data in 14,845 participants in the SAMINOR 1 Survey (2003—2004)

	Complete case (N=12,815)	Missing (N=2030)	Total (N=14,845)	p-value
**Age**	53.57 (10.74)	58.39 (11.73)	54.23 (11.00)	<0.001[1]
**Women**	6517 (50.9%)	1254 (61.8%)	7771 (52.3%)	<0.001[2]
**Died during follow-up**	1534 (12.0%)	480 (23.6%)	2014 (13.6%)	<0.001[2]
**Cause of death**				0.069[2]
Malignant tumor	540 (35.2%)	137 (28.5%)	677 (33.6%)	
CVD	436 (28.4%)	141 (29.4%)	577 (28.6%)	
Respiratory	166 (10.8%)	56 (11.7%)	222 (11.0%)	
Other	365 (23.8%)	134 (27.9%)	499 (24.8%)	
Unknown	27 (1.8%)	12 (2.5%)	39 (1.9%)	
**Weekly alcohol consumption**				<0.001[2]
Weekly	3362 (26.2%)	243 (15.3%)	3605 (25.0%)	
Less than weekly	7536 (58.8%)	932 (58.5%)	8468 (58.8%)	
Never/not last year	1917 (15.0%)	417 (26.2%)	2334 (16.2%)	
Missing data	0	438	438	
**Sedentary in leisure-time**				0.042[2]
Sedentary	2976 (23.2%)	184 (26.0%)	3160 (23.4%)	
Light	7732 (60.3%)	430 (60.7%)	8162 (60.4%)	
Moderate-hard	2107 (16.4%)	94 (13.3%)	2201 (16.3%)	
Missing data	0	1322	1322	
**Education**	11.41 (3.89)	9.62 (3.42)	11.26 (3.88)	<0.001[2]
Missing data	0	881	881	
**Smoking status**				0.001[2]
Yes, currently	4003 (31.2%)	621 (32.7%)	4624 (31.4%)	

Yes, previously	4603 (35.9%)	603 (31.7%)	5206 (35.4%)	
Never	4209 (32.8%)	676 (35.6%)	4885 (33.2%)	
Missing data	0	130	130	
**Glucose, mmol/L**[a]	5.33 (4.91, 5.91)	5.50 (5.02, 6.20)	5.35 (4.93, 5.95)	<0.001[3]
Missing data	0	28	28	
**Triglycerides (mmol/L)**[a]	1.42 (1.01, 2.04)	1.48 (1.07, 2.14)	1.43 (1.02, 2.05)	<0.001[3]
Missing data	0	10	10	
**HDL cholesterol (mmol/L)**	1.37 (0.38)	1.38 (0.37)	1.37 (0.38)	0.622[1]
Missing data	0	9	9	
**Systolic BP (mmHg)**	131.25 (19.68)	136.45 (22.11)	131.96 (20.11)	<0.001[1]
Missing data	0	5	5	
**Diastolic BP (mmHg)**	75.19 (10.32)	75.88 (11.09)	75.28 (10.43)	0.006[1]
Missing data	0	5	5	
**Waist circumference (cm)**	89.67 (11.98)	91.00 (12.08)	89.85 (12.00)	<0.001[1]
Missing data	0	28	28	
**Height in cm**	167.59 (9.45)	163.99 (9.27)	167.10 (9.51)	<0.001[1]
**Weight (kg)**	77.57 (14.42)	75.72 (14.22)	77.32 (14.41)	<0.001[1]
**Angina pectoris**	736 (5.9%)	182 (9.8%)	918 (6.4%)	<0.001[2]
Missing data	436	173	609	
**Stroke**	256 (2.1%)	72 (3.9%)	328 (2.3%)	<0.001[2]
Missing data	457	196	653	
**Myocardial infarction**	440 (3.6%)	95 (5.2%)	535 (3.8%)	<0.001[2]
Missing data	425	187	612	
**Diabetes mellitus**	454 (3.7%)	135 (7.3%)	589 (4.1%)	<0.001[2]
Missing data	427	172	599	
**Blood pressure-lowering drug**	2683 (21.1%)	673 (33.9%)	3356 (22.8%)	<0.001[2]

Missing data	109	46	155	
**Cholesterol-lowering drug**	1713 (13.6%)	378 (19.6%)	2091 (14.4%)	<0.001[2]
Missing data	255	99	354	
**Glucose-lowering drug**	401 (3.2%)	111 (6.0%)	512 (3.6%)	<0.001[2]
Missing data	449	170	619	

HDL = high-density lipoprotein, CVD = cardiovascular disease.

Continuous variables are reported as mean (standard deviation) and categorical variables are given as frequency (percent), if not stated otherwise

[a]Median (first quartile, third quartile)

[1]Two-sample t-test with equal variance

[2]Pearson's χ^2 test

[3]Wilcoxon rank sum test

Supplementary Table 2. Sample characteristics in mean (standard deviation) or frequency (percent) according to abdominal obesity phenotypes in 6517 women in the SAMINOR Study (2003—2004)

	Metabolically healthy non-abdominal obesity (N=2600, 39.9%)	Metabolically unhealthy non-abdominal obesity (N=1374, 21.1%)	Metabolically healthy abdominal obesity (N=832, 12.8%)	Metabolically unhealthy abdominal obesity (N=1711, 26.2%)	Total (N=6517)	p-value
**Age (years)**	49.0 (9.3)	56.5 (10.7)	52.0 (9.9)	57.5 (10.9)	53.2 (10.8)	<0.001[1]
**Ethnicity**						0.077[2]
non-Sami	2038 (78.4%)	1084 (78.9%)	632 (76.0%)	1296 (75.7%)	5050 (77.5%)	
Sami	562 (21.6%)	290 (21.1%)	200 (24.0%)	415 (24.3%)	1467 (22.5%)	
**Smoking**						<0.001[2]
Yes, currently	891 (34.3%)	495 (36.0%)	211 (25.4%)	451 (26.4%)	2048 (31.4%)	
Yes, previously	794 (30.5%)	368 (26.8%)	308 (37.0%)	592 (34.6%)	2062 (31.6%)	
Never	915 (35.2%)	511 (37.2%)	313 (37.6%)	668 (39.0%)	2407 (36.9%)	
**Died during follow-up**	119 (4.6%)	170 (12.4%)	42 (5.0%)	265 (15.5%)	596 (9.1%)	<0.001[2]
**Cause of death**						<0.001[2]
Malignant tumor	61 (51.3%)	51 (30.0%)	24 (57.1%)	82 (30.9%)	218 (36.6%)	
CVD	16 (13.4%)	48 (28.2%)	5 (11.9%)	83 (31.3%)	152 (25.5%)	
Respiratory	15 (12.6%)	18 (10.6%)	6 (14.3%)	23 (8.7%)	62 (10.4%)	
Other	25 (21.0%)	51 (30.0%)	5 (11.9%)	74 (27.9%)	155 (26.0%)	
Unknown	2 (1.7%)	2 (1.2%)	2 (4.8%)	3 (1.1%)	9 (1.5%)	
**Alcohol consumption**						<0.001[2]
Weekly	690 (26.5%)	251 (18.3%)	185 (22.2%)	213 (12.4%)	1339 (20.5%)	
Less than weekly	1592 (61.2%)	793 (57.7%)	497 (59.7%)	1010 (59.0%)	3892 (59.7%)	

Never/not last year	318 (12.2%)	330 (24.0%)	150 (18.0%)	488 (28.5%)	1286 (19.7%)	
**Leisure-time physical activity**						<0.001[2]
Sedentary	497 (19.1%)	292 (21.3%)	206 (24.8%)	530 (31.0%)	1525 (23.4%)	
Light	1745 (67.1%)	931 (67.8%)	539 (64.8%)	1042 (60.9%)	4257 (65.3%)	
Moderate-hard	358 (13.8%)	151 (11.0%)	87 (10.5%)	139 (8.1%)	735 (11.3%)	
**Education (years)**	12.7 (3.8)	10.8 (3.8)	11.8 (4.1)	10.5 (3.9)	11.6 (4.0)	<0.001[1]
**General obesity**	60 (2.3%)	95 (6.9%)	450 (54.1%)	1155 (67.5%)	1760 (27.0%)	<0.001[2]
**Metabolic syndrome**	0 (0.0%)	591 (43.0%)	0 (0.0%)	1347 (78.7%)	1938 (29.7%)	<0.001[2]
**Hypertension**	600 (23.1%)	946 (68.9%)	277 (33.3%)	1351 (79.0%)	3174 (48.7%)	<0.001[2]
**Increased waist circumference**	945 (36.3%)	800 (58.2%)	832 (100.0%)	1711 (100.0%)	4288 (65.8%)	<0.001[2]
**Low HDL cholesterol**	361 (13.9%)	630 (45.9%)	158 (19.0%)	1031 (60.3%)	2180 (33.5%)	<0.001[2]
**Elevated triglycerides**	167 (6.4%)	631 (45.9%)	88 (10.6%)	1083 (63.3%)	1969 (30.2%)	<0.001[2]
**Hyperglycemia**	21 (0.8%)	106 (7.7%)	3 (0.4%)	253 (14.8%)	383 (5.9%)	<0.001[2]
**Stroke**	0 (0.0%)	50 (4.0%)	0 (0.0%)	55 (3.5%)	105 (1.7%)	<0.001[2]
Missing data	3	130	2	119	254	
**Angina pectoris**	0 (0.0%)	101 (8.1%)	0 (0.0%)	179 (11.2%)	280 (4.5%)	<0.001[2]
Missing data	3	129	2	111	245	
**Myocardial infarction**	0 (0.0%)	42 (3.4%)	0 (0.0%)	52 (3.3%)	94 (1.5%)	<0.001[2]
Missing data	3	123	2	122	250	
**Diabetes**	0 (0.0%)	64 (5.1%)	0 (0.0%)	170 (10.6%)	234 (3.7%)	<0.001[2]
Missing data	3	128	2	109	242	
**Blood pressure-lowering drug**	0 (0.0%)	511 (37.9%)	0 (0.0%)	831 (49.3%)	1342 (20.8%)	<0.001[2]
Missing data	3	26	2	24	55	
**Cholesterol-lowering drug**	0 (0.0%)	336 (25.3%)	0 (0.0%)	427 (26.3%)	763 (12.0%)	<0.001[2]
Missing data	3	45	2	90	140	
**Glucose-lowering drug**	0 (0.0%)	64 (5.0%)	0 (0.0%)	140 (8.9%)	204 (3.2%)	<0.001[2]

Missing data	3	89	2	140	234

HDL = high-density lipoprotein, CVD = cardiovascular disease.

Continuous variables are reported as mean (standard deviation) and categorical variables are given as frequency (percent). In the final sample, missing data existed only in pre-existing disease and drug variables; in categorisation of metabolic health status, missing was assumed "no", but frequencies of missing are shown in this table. It is evident that most people with missing nevertheless was categorised in an unhealthy group.

[1]One way analysis of variance

[2]Pearson's χ^2 test

Supplementary Table 3. Sample characteristics in mean (standard deviation) or frequency (percent) according to abdominal obesity phenotypes in 6298 men in the SAMINOR Study (2003—2004)

	Metabolically healthy non-abdominal obesity (N=2558, 40.6%)	Metabolically unhealthy non-abdominal obesity (N=2408, 38.3%)	Metabolically healthy abdominal obesity (N=297, 4.7%)	Metabolically unhealthy abdominal obesity (N=1035, 16.4%)	Total (N=6298)	p-value
**Age (years)**	51.3 (9.8)	55.8 (11.0)	53.0 (10.9)	56.8 (10.3)	54.0 (10.6)	<0.001[1]
**Ethnicity**						<0.001[2]
non-Sami	1931 (75.5%)	1824 (75.7%)	240 (80.8%)	839 (81.1%)	4834 (76.8%)	
Sami	627 (24.5%)	584 (24.3%)	57 (19.2%)	196 (18.9%)	1464 (23.2%)	
**Smoking**						<0.001[2]
Yes, currently	896 (35.0%)	733 (30.4%)	74 (24.9%)	252 (24.3%)	1955 (31.0%)	
Yes, previously	852 (33.3%)	1007 (41.8%)	126 (42.4%)	556 (53.7%)	2541 (40.3%)	
Never	810 (31.7%)	668 (27.7%)	97 (32.7%)	227 (21.9%)	1802 (28.6%)	
**Died during follow-up**	241 (9.4%)	430 (17.9%)	40 (13.5%)	227 (21.9%)	938 (14.9%)	<0.001[2]
**Cause of death**						<0.001[2]
Malignant tumor	104 (43.2%)	130 (30.2%)	13 (32.5%)	75 (33.0%)	322 (34.3%)	
CVD	47 (19.5%)	137 (31.9%)	15 (37.5%)	85 (37.4%)	284 (30.3%)	
Respiratory	29 (12.0%)	48 (11.2%)	6 (15.0%)	21 (9.3%)	104 (11.1%)	
Other	56 (23.2%)	110 (25.6%)	6 (15.0%)	38 (16.7%)	210 (22.4%)	
Unknown	5 (2.1%)	5 (1.2%)	0 (0.0%)	8 (3.5%)	18 (1.9%)	
**Alcohol consumption**						<0.001[2]
Weekly	914 (35.7%)	710 (29.5%)	99 (33.3%)	300 (29.0%)	2023 (32.1%)	
Less than weekly	1453 (56.8%)	1400 (58.1%)	169 (56.9%)	622 (60.1%)	3644 (57.9%)	
Never/not last year	191 (7.5%)	298 (12.4%)	29 (9.8%)	113 (10.9%)	631 (10.0%)	

**Leisure-time physical activity**						<0.001[2]
Sedentary	513 (20.1%)	532 (22.1%)	85 (28.6%)	321 (31.0%)	1451 (23.0%)	
Light	1352 (52.9%)	1376 (57.1%)	161 (54.2%)	586 (56.6%)	3475 (55.2%)	
Moderate-hard	693 (27.1%)	500 (20.8%)	51 (17.2%)	128 (12.4%)	1372 (21.8%)	
**Education (years)**	11.8 (3.7)	10.8 (3.7)	11.1 (3.2)	10.7 (3.8)	11.2 (3.8)	<0.001[1]
**General obesity**	145 (5.7%)	326 (13.5%)	208 (70.0%)	804 (77.7%)	1483 (23.5%)	<0.001[2]
**Metabolic syndrome**	0 (0.0%)	1031 (42.8%)	0 (0.0%)	839 (81.1%)	1870 (29.7%)	<0.001[2]
**Hypertension**	939 (36.7%)	1915 (79.5%)	145 (48.8%)	901 (87.1%)	3900 (61.9%)	<0.001[2]
**Increased waist circumference**	670 (26.2%)	1093 (45.4%)	297 (100.0%)	1035 (100.0%)	3095 (49.1%)	<0.001[2]
**Low HDL cholesterol**	100 (3.9%)	804 (33.4%)	12 (4.0%)	444 (42.9%)	1360 (21.6%)	<0.001[2]
**Elevated triglycerides**	438 (17.1%)	1527 (63.4%)	67 (22.6%)	741 (71.6%)	2773 (44.0%)	<0.001[2]
**Hyperglycemia**	17 (0.7%)	253 (10.5%)	2 (0.7%)	168 (16.2%)	440 (7.0%)	<0.001[2]
**Stroke**	0 (0.0%)	103 (4.6%)	0 (0.0%)	48 (4.9%)	151 (2.5%)	<0.001[2]
Missing data	6	148	0	49	203	
**Angina pectoris**	0 (0.0%)	313 (13.8%)	0 (0.0%)	143 (14.4%)	456 (7.5%)	<0.001[2]
Missing data	6	142	0	43	191	
**Myocardial infarction**	0 (0.0%)	229 (10.0%)	0 (0.0%)	117 (11.8%)	346 (5.7%)	<0.001[2]
Missing data	6	128	0	41	175	
**Diabetes**	0 (0.0%)	124 (5.5%)	0 (0.0%)	96 (9.7%)	220 (3.6%)	<0.001[2]
Missing data	6	138	0	41	185	
**Blood pressure-lowering drug**	0 (0.0%)	858 (36.2%)	0 (0.0%)	483 (47.1%)	1341 (21.5%)	<0.001[2]
Missing data	6	38	0	10	54	
**Cholesterol-lowering drug**	0 (0.0%)	642 (27.6%)	0 (0.0%)	308 (30.6%)	950 (15.4%)	<0.001[2]
Missing data	6	79	0	30	115	
**Glucose-lowering drug**	0 (0.0%)	124 (5.5%)	0 (0.0%)	73 (7.5%)	197 (3.2%)	<0.001[2]
Missing data	6	142	0	67	215	

HDL = high-density lipoprotein, CVD = cardiovascular disease.

Continuous variables are reported as mean (standard deviation) and categorical variables are given as frequency (percent). In the final sample, missing data existed only in pre-existing disease and drug variables; in categorisation of metabolic health status, missing was assumed “no”, but frequencies of missing are shown in this table. It is evident that most people with missing nevertheless was categorised in an unhealthy group.

[1]One way analysis of variance

[2]Pearson’s χ^2 test

Supplementary Table 4. Sensitivity analyses. Hazard ratio (HR) and 95% confidence interval (CI) of metabolic syndrome (MetS), general and abdominal obesity phenotypes for all-cause mortality and CVD mortality in various samples of women in the SAMINOR 1 Survey (2003–2004)

		**All-cause mortality**						**CVD mortality**					
		Model 1		Model 2		Model 3		Model 1		Model 2		Model 3	
		HR	95% CI	HR	95% CI	HR	95% CI	HR	95% CI	HR	95% CI	HR	95% CI
Restricted to participants without pre-existing disease or receiving treatment for it (N=4601)	**MetS**												
	No	Ref.		Ref.		Ref.		Ref.		Ref.		Ref.	
	Yes	0.95	0.71 – 1.27	0.93	0.69 – 1.25	0.90	0.67 – 1.22	1.65	0.82 – 3.32	1.59	0.79 – 3.19	1.51	0.75 – 3.06
	**GOP**												
	MHNO	Ref.		Ref.		Ref.		Ref.		Ref.		Ref.	
	MUNO	0.68	0.45 – 1.03	0.66	0.44 – 1.00	0.63	0.42 – 0.95	1.11	0.41 – 3.04	1.01	0.37 – 2.79	0.92	0.33 – 2.56
	MHO	0.63	0.41 – 0.97	0.68	0.44 – 1.06	0.63	0.40 – 0.98	1.05	0.38 – 2.91	1.10	0.39 – 3.05	1.01	0.35 – 2.91
	MUO	1.13	0.78 – 1.66	1.19	0.81 – 1.73	1.12	0.76 – 1.65	2.46	1.05 – 5.78	2.58	1.10 – 6.06	2.51	1.06 – 5.98
	**AOP**												
	MHNAO	Ref.		Ref.		Ref.		Ref.		Ref.		Ref.	
	MUNAO	0.93	0.64 – 1.36	0.89	0.61 – 1.31	0.88	0.60 – 1.29	0.51	0.15 – 1.76	0.49	0.14 – 1.69	0.46	0.13 – 1.59
	MHAO	0.71	0.49 – 1.02	0.76	0.53 – 1.09	0.71	0.49 – 1.03	0.53	0.19 – 1.45	0.56	0.20 – 1.55	0.52	0.18 – 1.48
	MUAO	1.05	0.74 – 1.50	1.08	0.76 – 1.54	1.02	0.71 – 1.46	1.65	0.75 – 3.65	1.67	0.75 – 3.70	1.57	0.70 – 3.52

		**All-cause mortality**						**CVD mortality**					
		Model 1		Model 2		Model 3		Model 1		Model 2		Model 3	
		HR	95% CI	HR	95% CI	HR	95% CI	HR	95% CI	HR	95% CI	HR	95% CI
Restricted to participants who have never smoked (N=2407)	**MetS**												
	No	Ref.				Ref.		Ref.				Ref.	
	Yes	1.47	1.12 – 1.92			1.41	1.07 – 1.85	1.83	1.11 – 3.01			1.77	1.07 – 2.94
	**GOP**												
	MHNO	Ref.				Ref.		Ref.				Ref.	
	MUNO	1.40	0.95 – 2.08			1.36	0.91 – 2.03	2.95	1.12 – 7.77			2.83	1.06 – 7.52

	MHO	0.58	0.26 – 1.30			0.53	0.23 – 1.19	1.66	0.39 – 7.00			1.48	0.35 – 6.33
	MUO	1.56	1.05 – 2.31			1.40	0.93 – 2.11	3.39	1.29 – 8.91			2.97	1.10 – 8.02
	**AOP**												
	MHNAO	Ref.				Ref.		Ref.				Ref.	
	MUNAO	1.59	1.02 – 2.48			1.58	1.00 – 2.49	2.15	0.80 – 5.82			2.02	0.73 – 5.54
	MHAO	0.73	0.38 – 1.42			0.69	0.35 – 1.34	0.83	0.20 – 3.50			0.74	0.17 – 3.15
	MUAO	1.50	0.97 – 2.31			1.36	0.87 – 2.13	2.42	0.93 – 6.29			2.10	0.79 – 5.61

		**All-cause mortality**						**CVD mortality**					
		Model 1		Model 2		Model 3		Model 1		Model 2		Model 3	
		HR	95% CI	HR	95% CI	HR	95% CI	HR	95% CI	HR	95% CI	HR	95% CI
Original sample size (N=6517), but MetS categorised using conservative cut-offs for waist circumference, triglycerides and glucose	**MetS**												
	No	Ref.		Ref.		Ref.		Ref.		Ref.		Ref.	
	Yes	1.33	1.12 – 1.58	1.33	1.12 – 1.59	1.29	1.08 – 1.54	1.74	1.25 – 2.41	1.72	1.24 – 2.39	1.66	1.19 – 2.31
	**GOP**												
	MHNO	Ref.		Ref.		Ref.		Ref.		Ref.		Ref.	
	MUNO	1.32	1.07 – 1.62	1.33	1.08 – 1.64	1.31	1.06 – 1.61	3.38	2.00 – 5.73	3.41	2.01 – 5.77	3.33	1.96 – 5.66
	MHO	0.80	0.56 – 1.15	0.86	0.60 – 1.23	0.81	0.56 – 1.16	1.47	0.64 – 3.38	1.51	0.66 – 3.49	1.44	0.62 – 3.34
	MUO	1.26	1.01 – 1.57	1.38	1.10 – 1.72	1.28	1.02 – 1.61	3.03	1.76 – 5.21	3.16	1.83 – 5.44	2.90	1.66 – 5.07
	**AOP**												
	MHNAO	Ref.		Ref.		Ref.		Ref.		Ref.		Ref.	
	MUNAO	1.12	0.88 – 1.42	1.13	0.89 – 1.44	1.13	0.89 – 1.43	1.98	1.14 – 3.44	2.00	1.15 – 3.48	1.95	1.12 – 3.40
	MHAO	0.78	0.57 – 1.07	0.83	0.61 – 1.14	0.80	0.58 – 1.09	0.64	0.27 – 1.54	0.67	0.28 – 1.60	0.63	0.26 – 1.51
	MUAO	1.23	0.99 – 1.53	1.30	1.04 – 1.62	1.22	0.97 – 1.53	2.30	1.37 – 3.88	2.36	1.40 – 3.98	2.18	1.28 – 3.71

		**All-cause mortality**						**CVD mortality**					
		Model 1		Model 2		Model 3		Model 1		Model 2		Model 3	
		HR	95% CI	HR	95% CI	HR	95% CI	HR	95% CI	HR	95% CI	HR	95% CI
Multiply imputed data (m=20) of N=7771 women eligible for analysis	**MetS**												
	No	Ref.		Ref.		Ref.		Ref.		Ref.		Ref.	
	Yes	1.18	1.03 – 1.35	1.18	1.03 – 1.36	1.14	1.00 – 1.31	1.54	1.19 – 2.00	1.53	1.18 – 1.99	1.46	1.12 – 1.90
	**GOP**												
	MHNO	Ref.		Ref.		Ref.		Ref.		Ref.		Ref.	
	MUNO	1.19	1.00 – 1.42	1.20	1.02 – 1.43	1.18	0.98 – 1.41	2.50	1.63 – 3.84	2.50	1.63 – 3.84	2.45	1.59 – 3.77
	MHO	0.64	0.44 – 0.92	0.69	0.48 – 1.00	0.66	0.45 – 0.95	1.15	0.53 – 2.52	1.20	0.55 – 2.63	1.14	0.52 – 2.50

MUO	1.20	1.00 – 1.44	1.31	1.09 – 1.58	1.21	1.00 – 1.47	2.74	1.77 – 4.23	2.88	1.86 – 4.46	2.59	1.66 – 4.06
**AOP**												
MHNAO	Ref.		Ref.		Ref.		Ref.		Ref.		Ref.	
MUNAO	1.21	0.99 – 1.49	1.23	1.00 – 1.51	1.21	0.99 – 1.49	2.28	1.39 – 3.73	2.29	1.40 – 3.76	2.23	1.36 – 3.67
MHAO	0.75	0.56 – 1.01	0.80	0.60 – 1.08	0.77	0.57 – 1.03	1.16	0.58 – 2.30	1.21	0.60 – 2.40	1.12	0.56 – 2.25
MUAO	1.26	1.05 – 1.53	1.34	1.11 – 1.62	1.26	1.03 – 1.53	2.65	1.66 – 4.24	2.74	1.71 – 4.38	2.49	1.54 – 4.02

CVD = cardiovascular disease, HR = hazard ratio, CI = confidence interval. MetS = metabolic syndrome, ref. = reference, GOP = general obesity phenotypes, AOP = abdominal obesity phenotypes, MHNO = metabolically healthy non-obesity, MUNO = metabolically unhealthy non-obesity, MHO = metabolically unhealthy obesity, MUO = metabolically unhealthy obesity, MHNAO = metabolically healthy non-obesity, MUNAO = metabolically unhealthy non-abdominal obesity, MHAO = metabolically healthy abdominal obesity, MUAO = metabolically unhealthy abdominal obesity.

Model 1 is the crude model (all models inherently adjusted for age by using attained age as the time-scale). Model 2 was additionally adjusted for smoking, and model 3 was additionally adjusted for leisure-time physical activity, education and alcohol consumption (model 3). In all-cause mortality models, we applied stratified Cox with separate baseline hazards for subgroups of smoking status to satisfy the proportional hazard assumption.

Supplementary Table 5. Sensitivity analyses. Hazard ratio (HR) and 95% confidence interval (CI) of metabolic syndrome (MetS), general and abdominal obesity phenotypes for all-cause mortality and CVD mortality in various samples of men in the SAMINOR 1 Survey (2003–2004)

		All-cause mortality						CVD mortality					
		Model 1		Model 2		Model 3		Model 1		Model 2		Model 3	
		HR	95% CI	HR	95% CI	HR	95% CI	HR	95% CI	HR	95% CI	HR	95% CI
Restricted to participants without pre-existing disease or receiving treatment for it (N=4383)	**MetS**												
	No	Ref.		Ref.		Ref.		Ref.		Ref.		Ref.	
	Yes	1.05	0.84 – 1.31	1.09	0.87 – 1.36	1.06	0.84 – 1.33	1.16	0.74 – 1.83	1.27	0.80 – 2.01	1.21	0.76 – 1.91
	**GOP**												
	MHNO	Ref.		Ref.		Ref.		Ref.		Ref.		Ref.	
	MUNO	0.99	0.74 – 1.31	1.01	0.76 – 1.35	0.99	0.75 – 1.33	1.01	0.53 – 1.92	1.11	0.58 – 2.13	1.08	0.56 – 2.07
	MHO	1.14	0.82 – 1.59	1.28	0.91 – 1.80	1.22	0.87 – 1.72	2.71	1.59 – 4.63	3.29	1.91 – 5.68	3.09	1.78 – 5.36
	MUO	1.17	0.85 – 1.60	1.30	0.94 – 1.78	1.23	0.89 – 1.70	1.93	1.08 – 3.48	2.30	1.27 – 4.18	2.10	1.15 – 3.83
	**AOP**												
	MHNAO	Ref.		Ref.		Ref.		Ref.		Ref.		Ref.	
	MUNAO	1.13	0.90 – 1.42	1.18	0.94 – 1.49	1.16	0.92 – 1.46	1.36	0.84 – 2.20	1.48	0.91 – 2.39	1.44	0.89 – 2.33
	MHAO	1.14	0.82 – 1.60	1.26	0.90 – 1.77	1.21	0.86 – 1.71	2.19	1.22 – 3.93	2.48	1.37 – 4.47	2.40	1.32 – 4.35
	MUAO	1.27	0.93 – 1.73	1.35	0.99 – 1.85	1.30	0.95 – 1.78	1.49	0.77 – 2.87	1.68	0.87 – 3.25	1.59	0.82 – 3.09

		All-cause mortality						CVD mortality					
		Model 1		Model 2		Model 3		Model 1		Model 2		Model 3	
		HR	95% CI	HR	95% CI	HR	95% CI	HR	95% CI	HR	95% CI	HR	95% CI
Restricted to participants who have never smoked (N=1802)	**MetS**												
	No	Ref.				Ref.		Ref.				Ref.	
	Yes	1.10	0.79 – 1.53			1.03	0.73 – 1.44	1.30	0.75 – 2.27			1.22	0.68 – 2.18
	**GOP**												
	MHNO	Ref.				Ref.		Ref.				Ref.	
	MUNO	1.25	0.88 – 1.78			1.23	0.85 – 1.77	1.79	0.92 – 3.49			1.83	0.92 – 3.63
	MHO	1.95	0.92 – 4.12			1.85	0.87 – 3.91	3.84	1.24 – 11.89			3.78	1.21 – 11.80
	MUO	1.47	0.95 – 2.29			1.35	0.86 – 2.12	2.16	0.98 – 4.76			2.09	0.92 – 4.75
	**AOP**												
	MHNAO	Ref.				Ref.		Ref.				Ref.	

	MUNAO	1.48	1.02 – 2.15			1.46	1.00 – 2.13	2.81	1.28 – 6.18			3.00	1.35 – 6.68
	MHAO	1.39	0.65 – 2.96			1.37	0.64 – 2.93	3.52	1.05 – 11.77			3.95	1.17 – 13.39
	MUAO	1.62	1.01 – 2.61			1.47	0.89 – 2.42	3.23	1.29 – 8.09			3.20	1.22 – 8.38
		**All-cause mortality**						**CVD mortality**					
		Model 1		Model 2		Model 3		Model 1		Model 2		Model 3	
		HR	95% CI	HR	95% CI	HR	95% CI	HR	95% CI	HR	95% CI	HR	95% CI
Original	**MetS**												
sample size	No	Ref.		Ref.		Ref.		Ref.		Ref.		Ref.	
(N=6298), but	Yes	1.21	1.03 – 1.42	1.25	1.06 – 1.46	1.23	1.05 – 1.44	1.60	1.23 – 2.09	1.67	1.28 – 2.18	1.64	1.25 – 2.14
MetS	**GOP**												
categorised	MHNO	Ref.		Ref.		Ref.		Ref.		Ref.		Ref.	
using	MUNO	1.18	1.01 – 1.37	1.23	1.06 – 1.43	1.21	1.04 – 1.41	2.24	1.64 – 3.06	2.38	1.74 – 3.26	2.36	1.72 – 3.23
conservative	MHO	1.15	0.86 – 1.54	1.28	0.95 – 1.72	1.26	0.94 – 1.69	2.59	1.59 – 4.23	2.90	1.77 – 4.75	2.84	1.73 – 4.65
cut-offs for	MUO	1.25	1.05 – 1.50	1.40	1.17 – 1.69	1.36	1.13 – 1.64	2.58	1.83 – 3.65	3.01	2.12 – 4.28	2.90	2.04 – 4.14
waist	**AOP**												
circumference,	MHNAO	Ref.		Ref.		Ref.		Ref.		Ref.		Ref.	
triglycerides	MUNAO	1.20	1.02 – 1.40	1.25	1.07 – 1.46	1.23	1.05 – 1.44	1.94	1.41 – 2.68	2.08	1.50 – 2.87	2.04	1.48 – 2.83
and glucose	MHAO	1.23	0.91 – 1.66	1.33	0.98 – 1.80	1.30	0.96 – 1.76	1.93	1.10 – 3.38	2.10	1.20 – 3.68	2.03	1.16 – 3.57
	MUAO	1.42	1.18 – 1.70	1.55	1.29 – 1.86	1.51	1.25 – 1.81	2.73	1.93 – 3.86	3.08	2.17 – 4.38	2.97	2.08 – 4.24
		**All-cause mortality**						**CVD mortality**					
		Model 1		Model 2		Model 3		Model 1		Model 2		Model 3	
		HR	95% CI	HR	95% CI	HR	95% CI	HR	95% CI	HR	95% CI	HR	95% CI
Multiply	**MetS**												
imputed data	No	Ref.		Ref.		Ref.		Ref.		Ref.		Ref.	
(m=20) of	Yes	1.09	0.96 – 1.23	1.13	1.00 – 1.28	1.11	0.98 – 1.26	1.46	1.18 – 1.81	1.54	1.24 – 1.91	1.50	1.20 – 1.87
N=7074	**GOP**												
participants	MHNO	Ref.		Ref.		Ref.		Ref.		Ref.		Ref.	
eligible for	MUNO	1.14	0.99 – 1.31	1.21	1.05 – 1.39	1.18	1.02 – 1.36	1.81	1.36 – 2.40	1.87	1.38 – 2.54	1.88	1.40 – 2.51
analysis	MHO	1.12	0.83 – 1.52	1.27	0.94 – 1.72	1.23	0.91 – 1.66	2.42	1.48 – 3.94	2.30	1.38 – 3.83	2.57	1.56 – 4.21
	MUO	1.21	1.03 – 1.43	1.39	1.17 – 1.64	1.33	1.12 – 1.58	2.36	1.73 – 3.22	2.74	1.98 – 3.81	2.61	1.89 – 3.60
	**AOP**												
	MHNAO	Ref.		Ref.		Ref.		Ref.		Ref.		Ref.	
	MUNAO	1.12	0.97 – 1.30	1.20	1.03 – 1.39	1.17	1.00 – 1.35	1.73	1.28 – 2.35	1.87	1.38 – 2.54	1.78	1.31 – 2.42

MHAO	1.15	0.86 – 1.54	1.26	0.94 – 1.69	1.21	0.90 – 1.63	2.11	1.27 – 3.52	2.30	1.38 – 3.83	2.12	1.27 – 3.55
MUAO	1.34	1.13 – 1.58	1.49	1.26 – 1.76	1.43	1.21 – 1.70	2.44	1.76 – 3.37	2.74	1.98 – 3.81	2.60	1.87 – 3.63

CVD = cardiovascular disease, HR = hazard ratio, CI = confidence interval. MetS = metabolic syndrome, ref. = reference, GOP = general obesity phenotypes, AOP = abdominal obesity phenotypes, MHNO = metabolically healthy non-obesity, MUNO = metabolically unhealthy non-obesity, MHO = metabolically unhealthy obesity, MUO = metabolically unhealthy obesity, MHNAO = metabolically healthy non-obesity, MUNAO = metabolically unhealthy non-abdominal obesity, MHAO = metabolically healthy abdominal obesity, MUAO = metabolically unhealthy abdominal obesity.

Model 1 is the crude model (all models inherently adjusted for age by using attained age as the time-scale). Model 2 was additionally adjusted for smoking, and model 3 was additionally adjusted for leisure-time physical activity, education and alcohol consumption (model 3). In all-cause mortality models, we applied stratified Cox with separate baseline hazards for subgroups of smoking status to satisfy the proportional hazard assumption.

Supplementary Table 6. All-cause and CVD mortality according to MetS, general and abdominal obesity phenotypes: Hazard ratios (HR) and 95% confidence intervals (CI) from Cox proportional hazards models of 12,815 men and women in SAMINOR 1 (2003–2004)

				Model 1		Model 2		Model 3	
	**Cases**	**Person-years**	**IR**	**HR**	**95% CI**	**HR**	**95% CI**	**HR**	**95% CI**
**Outcome: All-cause mortality**									
**Metabolic syndrome**									
No	970	133,629.1	7.3	Ref.		Ref.		Ref.	
Yes	564	55,729.5	10.1	1.10	0.99 – 1.22	1.12	1.01 – 1.25	1.10	0.99 – 1.23
**General obesity phenotypes**									
Metabolically healthy non-obese	451	90,864.1	5.0	Ref.		Ref.		Ref.	
Metabolically unhealthy non-obese	632	50,808.6	12.4	1.13	1.00 – 1.28	1.16	1.03 – 1.31	1.14	1.00 – 1.29
Metabolically healthy obese	64	13,135.3	4.9	0.87	0.67 – 1.13	0.95	0.73 – 1.24	0.92	0.71 – 1.20
Metabolically unhealthy obese	387	34,550.6	11.2	1.20	1.05 – 1.38	1.33	1.15 – 1.53	1.26	1.10 – 1.46
**Abdominal obesity phenotypes**									
Metabolically healthy non-abdominally obese	360	77,437.9	4.6	Ref.		Ref.		Ref.	
Metabolically unhealthy non-abdominally obese	600	55,204.6	10.9	1.13	0.99 – 1.29	1.17	1.03 – 1.34	1.15	1.01 – 1.32
Metabolically healthy abdominally obese	82	16,915.5	4.8	0.88	0.69 – 1.12	0.95	0.74 – 1.21	0.92	0.72 – 1.17
Metabolically unhealthy abdominally obese	492	39,800.6	12.4	1.33	1.15 – 1.53	1.43	1.24 – 1.65	1.36	1.18 – 1.58
**Outcome: CVD mortality**									
**Metabolic syndrome**									
No	243	133,629.1	1.8	Ref.		Ref.		Ref.	
Yes	193	55,729.5	3.5	1.47	1.22 – 1.78	1.51	1.25 – 1.83	1.48	1.22 – 1.79
**General obesity phenotypes**									
Metabolically healthy non-obese	72	90,864.1	0.8	Ref.		Ref.		Ref.	
Metabolically unhealthy non-obese	208	50,808.6	4.1	2.18	1.66 – 2.86	2.30	1.75 – 3.02	2.24	1.70 – 2.95
Metabolically healthy obese	23	13,135.3	1.8	1.96	1.22 – 3.14	2.16	1.34 – 3.48	2.06	1.28 – 3.33
Metabolically unhealthy obese	133	34,550.6	3.8	2.48	1.85 – 3.32	2.78	2.07 – 3.75	2.61	1.93 – 3.53
**Abdominal obesity phenotypes**									
Metabolically healthy non-abdominally obese	63	77,437.9	0.8	Ref.		Ref.		Ref.	
Metabolically unhealthy non-abdominally obese	185	55,204.6	3.4	1.84	1.37 – 2.45	1.95	1.46 – 2.61	1.90	1.42 – 2.54
Metabolically healthy abdominally obese	20	16,915.5	1.2	1.27	0.76 – 2.10	1.36	0.82 – 2.27	1.30	0.78 – 2.17

Metabolically unhealthy abdominally obese	168	39,800.6	4.2	2.50	1.85 – 3.36	2.74	2.03 – 3.71	2.58	1.90 – 3.51

HR = hazard ratio, CI = confidence interval, IR = crude incidence rate per 1000 person-years.
Adjustments were made for sex (model 1) plus smoking (model 2) plus leisure-time physical activity, education and alcohol consumption (model 3).
All models were inherently adjusted for age by using attained age as the time-scale. In the all-cause mortality models, we applied stratified Cox models with separate baseline hazards for subgroups of smoking status to satisfy the proportional hazard assumption. In the CVD mortality models, we applied stratified Cox models with separate baseline hazards for subgroups of sex and smoking status to satisfy the proportional hazard assumption.

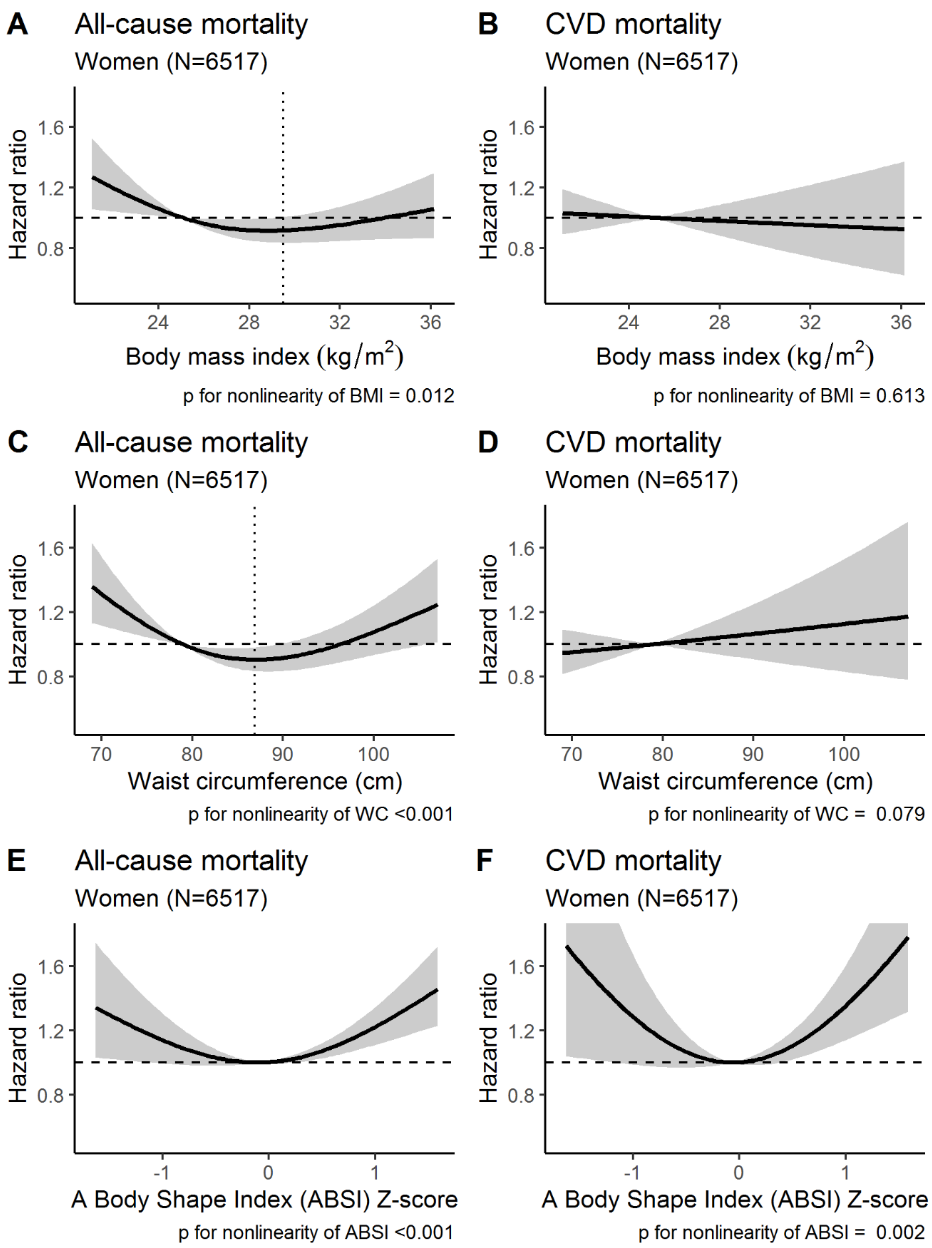

Supplementary Figure 1. The functional relationships between mortality (all-cause and CVD) and continuous obesity measures (body mass index, waist circumference and a body shape index) with corresponding hazard ratios with 95% confidence bands in women. The reference of all curves were women with a BMI of 26.7 kg/m^2, a waist circumference of 79 cm and a body shape index Z-score of 0 (median values). P-values originates from likelihood ratio tests comparing models with/without linear terms terms. Estimates are predicted for median values of confounders (smoking, leisure-time physical activity, education, alcohol consumption). All models were inherently adjusted for age by using attained age as the time-scale. The vertical, dotted lines represent the nadir of risk. ABSI = a body shape index, BMI = body mass index, WC = waist circumference.

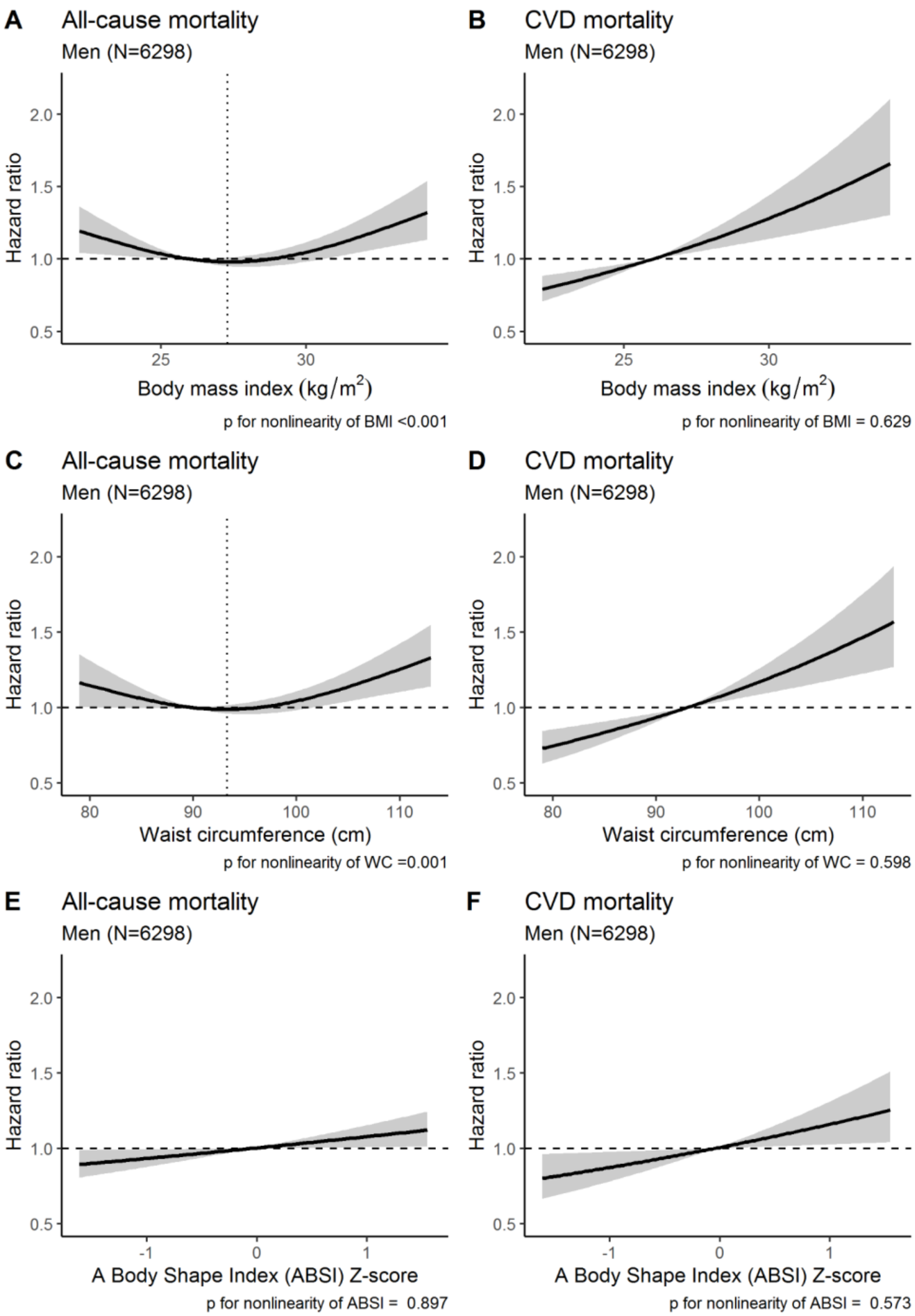

Supplementary Figure 2. The functional relationships between mortality (all-cause and CVD) and continuous obesity measures (body mass index, waist circumference and a body shape index) with corresponding hazard ratios with 95% confidence bands in men. The reference of all curves were men with a BMI of 27.2, a waist circumference of 90 cm and a body shape index Z-score of 0 (median values). P-values originates from likelihood ratio tests comparing models with/without linear terms terms. Estimates are predicted for median values of confounders (smoking, leisure-time physical activity, education, alcohol consumption). All models were inherently adjusted for age by using attained age as the time-scale. The vertical, dotted lines represent the nadir of risk. ABSI = a body shape index, BMI = body mass index, WC = waist circumference.
